# Systematic review of methodology used in clinical studies evaluating the benefits of proton beam therapy

**DOI:** 10.1016/j.ctro.2019.07.002

**Published:** 2019-07-12

**Authors:** Mercy Ofuya, Lucy McParland, Louise Murray, Sarah Brown, David Sebag-Montefiore, Emma Hall

**Affiliations:** a Clinical Trials and Statistics Unit at The Institute of Cancer Research, London, United Kingdom; b Clinical Trials Research Unit, Leeds Institute of Clinical Trials Research, University of Leeds, Leeds, United Kingdom; c Leeds Cancer Centre, Leeds Teaching Hospitals NHS Trust, Leeds, United Kingdom; d Leeds Institute of Molecular Research, University of Leeds, Leeds, United Kingdom

**Keywords:** Proton beam therapy, Randomized controlled trials, Clinical trial methodology, Patient reported outcomes, Systematic review

## Abstract

**Background:**

Proton beam therapy (PBT) delivers high-energy radiation to target tumours while sparing surrounding normal tissues. The dosimetric advantages of PBT over traditional photon radiotherapy may be clear but the translation of this benefit into clinically meaningful reductions in toxicities and improved quality-of-life (QoL) needs to be determined. Randomised controlled trials (RCTs) are considered the gold standard for generating the highest-level evidence in medicine. The objectives of this systematic review were to provide an overview of published clinical studies evaluating the benefits of PBT, and to examine the methodology used in clinical trials with respect to study design and outcomes.

**Methods:**

PubMed, EMBASE and Cochrane databases were systematically searched for published clinical studies where PBT was a cancer treatment intervention. All randomised and non-randomised studies, prospective or retrospective, were eligible for inclusion.

**Results:**

In total, 219 studies were included. Prospective studies comprised 89/219 (41%), and of these, the number of randomised phase II and III trials were 5/89 (6%) and 3/89 (3%) respectively. Of all the phase II and III trials, 18/24 (75%) were conducted at a single PBT centre. Over one-third of authors recommended an increase in length of follow up. Research design and/or findings were poorly reported in 74/89 (83%) of prospective studies. Patient reported outcomes were assessed in only 19/89 (21%) of prospective studies.

**Conclusions:**

Prospective randomised evidence for PBT is limited. The set-up of national PBT services in several countries provides an opportunity to guide the optimal design of prospective studies, including RCTs, to evaluate the benefits of PBT across various disease sites. Collaboration between PBT centres, both nationally and internationally, would increase potential for the generation of practice changing evidence. There is a need to facilitate and guide the collection and analysis of meaningful outcome data, including late toxicities and patient reported QoL.

## Highlights

• Less than half of published clinical studies of proton beam therapy are prospective.
• Only 10% of prospective studies of proton beam therapy are randomised.
• Toxicity is the most common primary endpoint of interventional studies.

## 1 Introduction

Advancements in the delivery of radiotherapy require improvements in efficacy by delivering an optimal dose to the target tumour while minimising dose to the surrounding normal tissues in an effort to reduce toxicity. Standard photon radiotherapy delivers a relatively high entrance dose before depositing energy in the target and then continuing to deposit energy beyond the target, albeit with a gradual reduction in dose. In contrast, proton beam therapy (PBT) is an advanced radiation treatment characterised by its Bragg peak, whereby a high dose is delivered to the target, followed by an immediate drop in energy resulting in minimum to no exit dose compared to standard photon radiotherapy, thereby sparing surrounding normal tissues. This phenomenon potentially increases the therapeutic ratio.

Evidence from dosimetric and planning studies show that lower mean radiation doses are delivered to surrounding normal tissues using protons compared to photons in the treatment of paediatric [1], [2], [3], [4], [5] and adult malignancies [6], [7], [8], [9], [10], [11]. Although these studies suggest a dosimetric advantage of PBT, it is uncertain whether this translates into measurable clinically significant benefits for the patient in the short and/or long term. Other concerns include range uncertainties, organ motion, and anatomical changes of target tumour or normal organ.

Past reviews on clinical outcomes criticise the lack of robust evidence of the clinical superiority of protons over photons [12], [13], [14], [15], and the debate on the need for randomisation in clinical trials evaluating the benefits of PBT persists [16], [17], [18], [19], [20], [21], [22], [23]. Nevertheless, uncertainties about the effect of protons on toxicity, tumour control, and survival, warrant prospective high quality studies. Wherever possible, randomised controlled trials are recommended as they are considered by most to be the gold standard for generating practice changing evidence [22], [24], [25].

An estimated 149,345 patients were treated with protons between 1954 and 2016 [26] and the development of new PBT centres in countries such as United Kingdom (UK), India, Australia, Norway and Denmark suggests a global increase in treatment uptake. Challenges in designing clinical trials for PBT include barriers to enrolment such as cost coverage (by governments or third party payers) [25], patient preference, and travel distance to PBT centres. However, findings from studies exploring patient involvement in proton trials suggest that patients would be willing to participate in PBT trials [27], [28].

This systematic review aims to provide an overview of published clinical studies evaluating the benefits of PBT. Specifically this review examines the methodology used in clinical trials with respect to study design and endpoints in an effort to highlight to a predominantly non-proton clinical audience both the weaknesses in the existing evidence and the need for high quality randomised trials.

## 2 Methods

### 2.1 Search strategy and selection criteria

Electronic systematic searches were conducted in PubMed, Embase and Cochrane databases for published clinical studies where PBT was a therapeutic intervention for cancer conditions. Search terms included proton beam therapy, cancer, carcinoma, and various anatomical regions and tumour sites. These terms were combined in accordance with the search platform for each database. The search was limited to human studies in PubMed and Embase but there were no date restrictions. The search strategy used in each database is presented in the supplementary appendix (A.1). The database search was conducted in August 2017, and was later updated in PubMed in July 2018.

Inclusion criteria were clinical studies where PBT was a therapeutic intervention for cancer, all comparative (randomised and non-randomised) and non-comparative studies having one or more treatment arms and prospective and retrospective studies. The latter were included in order to provide a comprehensive overview of study designs used in various disease sites indicated for PBT. Systematic reviews were included at the screening stage, and the main texts and references of these were searched in order to capture any further eligible primary studies that were not included in the database search, but were not included in this review.

Exclusion criteria included case reports, studies regarding PBT intervention for non-cancer conditions, histopathology, and those which predominantly focused on PBT treatment planning optimisation, dosimetry and techniques. Other exclusions were studies regarding cost-effectiveness of PBT, reports by health committees or government bodies, systematic reviews and non-clinical reviews on geographical distribution of PBT treatment centres and uptake. Abstracts and protocols were excluded, as these were not comparable with the full texts of eligible studies at the same level of detail. Although there were no language restrictions in the database search strategy, non-English language studies for which translations could not be accessed were excluded.

**Fig. 1:**
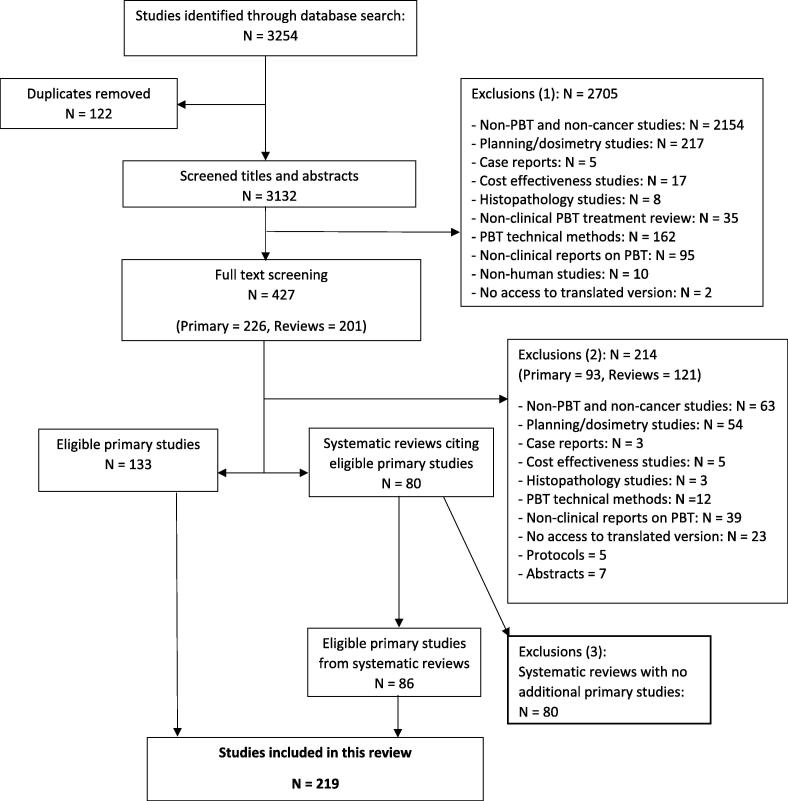
Flowchart of search strategy in accordance with PRISMA statement. Abbreviations: PBT Proton beam therapy; PRISMA = Preferred Reporting Items for Systematic Reviews and Meta-Analyses.

One reviewer conducted the database search and screened the articles for eligibility. A second reviewer independently screened a random sample (20% (44/219)) of articles to confirm eligibility. The reviewers discussed and resolved by consensus any disagreements on inclusion and exclusion of studies. Titles and abstracts were screened, the full texts of eligible studies were retrieved, and systematic reviews were searched for eligible studies. The search strategy is presented in Fig. 1 in accordance with the Preferred Reporting Items for Systematic Reviews and Meta-Analyses (PRISMA) statement [29].

### 2.2 Data extraction and synthesis

Data were extracted using a template in Microsoft Excel (Table A.1, supplementary appendix). Data are presented graphically and using descriptive summary statistics. All analyses were performed using Stata v13.0 [30].

## 3 Results

In total, 3254 articles were identified. After duplicates were removed, titles and abstracts of 3132 records were screened for eligibility. Eighty-six percent (n = 2705) of articles were excluded at this stage, the majority of which were non-PBT and non-cancer studies (n = 2154). Full texts of 427 articles (226 primary studies and 201 systematic reviews) were screened to further identify eligible studies. Of these, 219 peer reviewed studies (excluding abstracts and protocols) published between 1979 and 2018 were included.

**Fig. 2:**
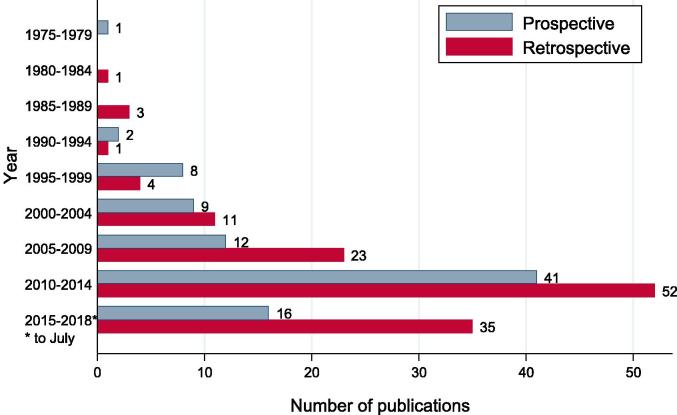
Distribution of prospective and retrospective studies by year of publication (n = 219).

Prospective and retrospective studies comprised 41% (89/219) and 59% (130/219) of studies respectively (Fig. 2). Retrospective data were obtained from patients’ medical records in 90% (117/130) of cases, cancer database/registry in 6% (8/130), or past prospective study data in 1.5% (2/130); sources were not stated in 3/130 (2%) studies. The median (range) sample size for retrospective studies was 53 (6–243,822) and 51 (3–1447) for prospective studies (phase I/feasibility/pilot: 20 (8–211); phase I/II: 25 (12–202); phase II: 59 (23–393); phase III: 188 (151–202); observational: 51 (3–1447)) (supplementary appendix Figs. A.1–A.2). Table A.2 (supplementary appendix) presents details of all studies included in this review.

**Fig. 3:**
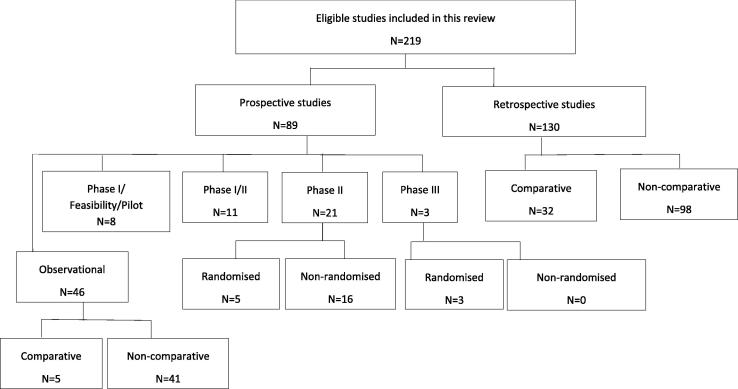
Distribution of study designs used in included studies.

Fig. 3 shows the distribution of various designs used in the included studies. Of the prospective studies, just 5/89 (6%) and 3/89 (3%) were randomised phase II and III trials respectively. Table 1 shows the details of RCTs, which involved PBT. The highest proportion of prospective studies were observational (52%; 46/89), and 11% (5/46) of these made comparisons between treatment arms. In three prospective studies (Sejpal 2011, Hoppe 2014 and Blanchard 2016), comparisons were made with historical control data on clinical outcomes of intensive modulated radiotherapy (IMRT).

**Table 1:** Details of phase II and III randomised controlled trials that involved PBT (n = 8).

Authors, year	Condition	Aim	RCT phase	Age range (years)	Sample size
Bush et al, 2016 [31]	Hepatocellular carcinoma	To report interim analyses of PBT versus transarterial chemo-embolization	Phase 2	Not stated	69
Desjardins et al, 2006 [32]	Uveal melanoma	To determine the effect of systematic transpupillary thermotherapy after PBT	Phase 3	22–88	151
Gragoudas et al, 2000 [33]	Choroidal melanoma	To determine the effect of a reduction in PBT dose from standard 70 CGE to 50 CGE on treatment outcomes	Phase 3	19–86	188
Habl et al, 2016 [34]	Prostate cancer	To explore the safety and feasibility of primary hypofractionated irradiation with PBT and carbon ions in a raster scan technique	Phase 2	40–80	92
Kim et al, 2013 [35]	Prostate cancer	To investigate the feasibility of hypofractionated PBT in treatment of prostate cancer	Phase 2	44–85	82
Liao et al, 2018 [36]	Non–small cell lung cancer	To compare outcomes of passive scattering PBT versus IMRT	Phase 3	33–85	149
Shipley et al, 1995 [37]	Prostate cancer	To evaluate the possible increased efficacy of a higher dose of radiation on the local recurrence rate and patient survival	Phase 3	46–85	202
Zietman et al, 2010 [38]	Prostate cancer	To test the hypothesis that increasing radiation dose improves clinical outcomes	Phase 2	45–91	393

*Abbreviations*: RCT = Randomised controlled trial; PBT = Proton beam therapy; IMRT = Intensive modulated radiotherapy; CGE = Cobalt gray equivalent.

**Fig. 4:**
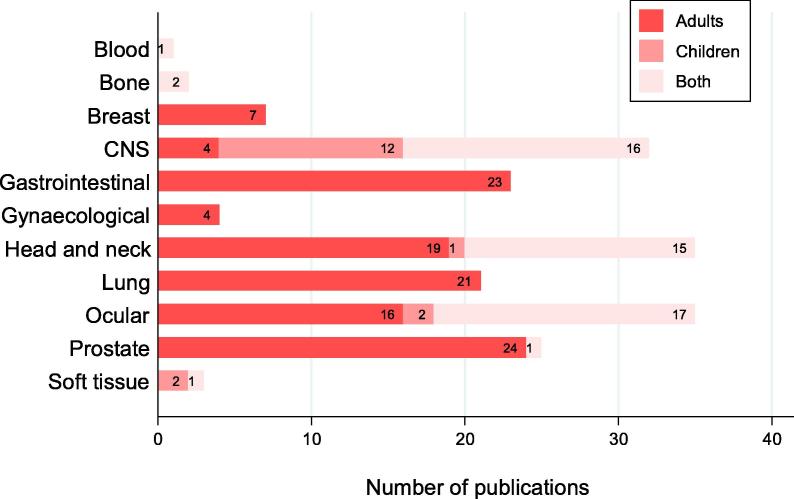
Distribution of disease sites in adult and paediatric patients (n = 188). Abbreviation: CNS = Central nervous system.

PBT was administered to adults, children or both in 54% (118/219), 8% (17/219) and 24% (53/219) of studies respectively and the ages of all patients ranged from one month to 95 years. Fig. 4 shows the distribution of treated tumour sites by adults and children. The ages of patients were not reported in 5% (12/219) of studies and it was not possible to determine the age range in 9% (19/219) of studies as these reported only the estimated mean or median.

**Fig. 5:**
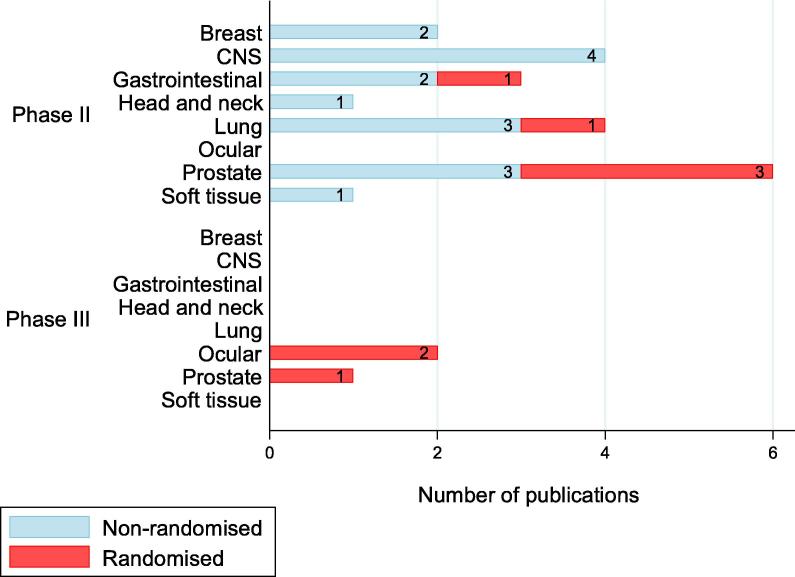
Distribution of phase II and III trials by disease site treated by PBT (n = 24). Abbreviation: CNS = Central nervous system.

Direct comparisons between treatment effects of PBT and photons were investigated in 7% (6/89) of prospective studies (Table A.3, supplementary appendix). Other cancer treatment interventions reported in prospective studies and compared with protons were chemotherapy, carbon ions, transarterial chemoembolization, and transpupillary thermotherapy. PBT alone or in combination with other therapies was administered in treatment of cancer of the breast (7/219), CNS (37/219), blood (1/219), bone (2/219), gynaecological organs (4/219), gastrointestinal tract (26/219), head and neck (40/219), eye (47/219), prostate (28/219) and soft tissue (3/219). Fig. 5 shows the distribution of phase II and III trials by disease site treated by PBT.

### 3.1 PBT centres – location and collaboration

**Fig. 6:**
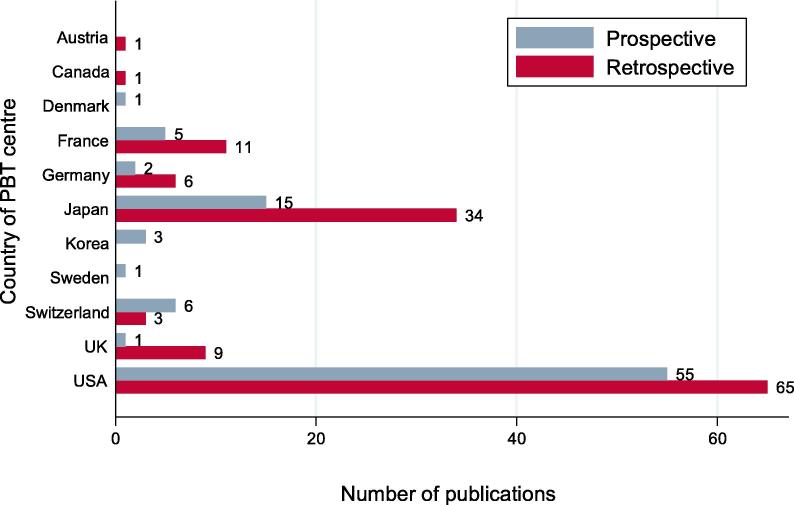
Distribution of geographical location of PBT treatment centre (n = 219). Abbreviation: PBT = Proton beam therapy.

A high proportion of prospective studies (90%; 80/89) and 75% (18/24) of phase II and III trials were conducted at single PBT centres. In most studies, it was not possible to ascertain patterns of referral to the PBT study centres. The countries with the highest number of published studies were the United States (120/219 studies; 55 prospective, 65 retrospective), Japan (49/219 studies; 15 prospective, 34 retrospective) and France (16/219 studies; 5 prospective, 11 retrospective) (Fig. 6).

### 3.2 Publications of prospective and retrospective studies

The highest proportion of studies were published between 2010 and 2014 (42% (93/219; 41 prospective, 52 retrospective)) (Fig. 2). The observed increase in number of publications between 2009 and 2014 is likely due to the development of new proton centres from 2006 particularly in United States [39]. Nineteen percent (25/130) of authors of retrospective studies made recommendations about the need for studies with prospective designs; the number of retrospective studies appears to have increased over time.

### 3.3 Outcomes

Patient reported outcome measures (PROMs), overall survival and clinician assessed acute and late toxicity rates were collected prospectively in 19 (21%), 55 (62%), 75 (84%) and 64 (72%) of the 89 prospective studies respectively either as primary or secondary endpoints.

A primary endpoint was not defined in 56% (24/43) interventional studies. Two of three phase III studies, 13/21 phase II studies, 3/11 phase I/II studies and 1/8 phase I/feasibility/pilot studies defined a primary endpoint (n = 16) and/or powered their studies for a specific outcome(s) (n = 3). Of the different types of primary endpoints defined, nine were toxicity related (acute (n = 4), late (n = 2), undefined assessment period (n = 3)), and four were efficacy related (progression-free survival (n = 2), overall survival (n = 1), local failure (n = 1)). Composite/co-primary endpoints were defined in six studies and these included toxicity and feasibility of treatment delivery (n = 2), toxicity and local recurrence/disease control (n = 3) and disease-free and overall survival (n = 1).

Acute and late toxicity criteria used were Common Terminology Criteria for Adverse Events (CTCAE), Radiation Therapy Oncology Group (RTOG) toxicity grading, Late Effects Normal Tissue Task Force (LENT), Late Effects Normal Tissue Task Force-Subjective, Objective, Management and Analytic (LENT-SOMA), and Brock ototoxicity grading scale. Sixteen different questionnaires were used to assess PROMs (supplementary appendix A1.4). The authors of a case matched study, which aimed to compare the clinical outcomes of IMRT and intensity modulated proton therapy (IMPT) in head and neck cancer patients, suggested that PROMs should be the measure of interest in proton studies [40].

### 3.4 Follow up post-treatment

Ninety percent (79/89) of prospective studies reported duration of follow up and this ranged from 4 days to 191 months (observational: 4 days − 191 months; phase I/feasibility/pilot: 2–97 months; phase I/II: 2–104 months; phase II: 2–150 months; phase III: 3 – 139 months). In 38% (34/89) of prospective studies, the authors recommended an increase in length of follow up in patients treated with PBT. In particular, 21% (19/89) of authors suggested the need to study long-term and late toxicities to enable assessment of the patterns and severity of these outcomes. In one study, the authors noted that clinical benefits of improved dosimetry of PBT based on toxicity assessments may not be evident in the first few years after treatment, and further observation may show meaningful differences in clinician and patient reported toxicity over time [41]. In addition, authors emphasized the need for longer follow up to enable assessment of late radiation sequelae that may occur many years after treatment [42] and the need to assess long term QoL outcomes data [43]. Gardner et al [44] noted the potential for recall and selective biases in the long-term assessment of QoL in patients treated with high dose radiation techniques.

### 3.5 Randomisation

Allocation to treatment arms in the randomised studies was by the simple randomisation method in 2/8 phase II/III studies and the stratified randomisation method in 4/8 studies. The Zelen design was used in one study and this involves randomising patients to treatment groups before obtaining consent. In a further RCT, patients were assigned to treatment groups by means of a Bayesian adaptive approach which relies on event information being updated in real time such that the ratio of allocation to treatment arms can be adjusted before the next patient is randomly assigned [36]. Intention-to-treat (ITT) analysis, i.e. analysing data according to the groups to which the patients were enrolled and randomised, was reported in 3/5 and 2/3 randomised phase II and III trials respectively, while the other RCTs (n = 3/8) did not state whether or not this approach was used.

### 3.6 Reporting

Eighty-seven percent (191/219) of studies reported eligibility criteria. Approximately 11% (24/219) of all included studies did not state the statistical methods used in analysing the study data and 66% (31/43) of interventional studies did not state the justification for the sample size used. It was not possible to determine the estimated proportion of studies that involved a methodologist because this information was not published in all studies. A high proportion (88%; 78/89) of prospective studies assessed acute (75/89) and/or late (64/89) toxicities as either primary or secondary outcomes. Of these, 22% (17/78) did not state the grading criteria/system used. In addition, there was no definition for timelines of acute and late toxicity assessment in 67% (50/75) and 72% (46/64) of studies respectively. Timeline definitions for acute toxicities ranged from 2 weeks to within 90 days after completion of radiotherapy treatment. For late toxicity, this was >90 days.

One prospective study (multicentre, phase II) investigating clinical outcomes from PBT in prostate cancer reported conducting quality assurance assessment [45]. However, it is unclear whether this was performed in other studies.

### 3.7 Limitations reported in retrospective studies

Thirty-eight percent (49/130) of retrospective studies reported limitations of this study design and these include the constraint on the types of data that can be extracted which may possibly impede the extraction of complete data [39]. The authors of a large retrospective study (n = 243,822), which compared protons with photons using data from the National Cancer Database (NCDB), stated that a major limitation was the lack of both acute and late toxicity data [46]. Other retrospective studies reported that this puts a restriction on the types of analysis that can be reliably performed such as the comparison of toxicity rates between PBT and photons, assessment of patient reported outcomes, and differences in periods of diagnosis and treatment.

Other limitations cited include the potential of biases, such as selection bias (e.g. exclusion of patients with short follow up), under- or over-reporting of specific outcomes or toxicities, small sizes of patient cohorts in case series. In addition, difficulty in comparing findings with those of past studies due to limited information about treatment regimen, differences in treatment protocols or modifications in treatment delivery technique.

### 3.8 Limitations reported in prospective studies

Of the randomised phase II and III trials, 4/8 studies reported limitations. These included small sample sizes, short follow up period and the lack of comparison with other conformal techniques in one study [38] which compared conventional dose with high dose. Similarly, short follow up was reported as a limitation in 2/8 phase I/feasibility/pilot studies. For 50% (8/16) of the non-randomised phase II studies, shortcomings were either limited availability of PBT centres due to high costs of set-up, short follow up, small sample sizes, the lack of a photon control arm, or differences in specifications of PBT centres between participating institutions. Other limitations were the heterogeneity in the patient population and inconsistency in the number of proton beam fields used across patients. In a phase I/II study, technical limitations such as machine time constraints and the use of a single beam rather than multiple beams were reported [47], and in a further study [48], comparison with retrospective data on IMRT was stated as a limitation.

## 4 Discussion

The aim of this systematic review was to provide an assessment of the methodology used in clinical studies investigating PBT with respect to study designs, outcomes, reporting of findings and limitations. As the development of PBT services increases, it is imperative to demonstrate and evaluate the key anticipated benefits, or lack of benefits of PBT. There is a need to maintain equipoise and restrict bias particularly as standard photon technologies are constantly improving resulting in more conformal techniques [48]. To our knowledge, this is the first systematic review of the methodology used in clinical studies evaluating the benefits of PBT. Several systematic reviews have assessed the landscape of ongoing/recently completed observational and/or interventional PBT trials including phase II/III trials on PBT [49], [50], [51]. Verma et al [52] reviewed patient reported outcomes, with a focus on QoL in patients treated with PBT, the implications of these on cost effectiveness for stakeholders, value based oncology (VBO), and trial design. As further prospective clinical studies are developed, robust methodology in trial design, analysis and reporting will be pivotal in influencing the clinical management of cancer patients treated with PBT.

Our findings show that there is very limited level 1 evidence for the use of PBT, as only 3% (3/89) of published proton studies were phase III randomised controlled trials, despite the steady increase in treatment uptake, across various tumour sites between 1979 and 2018. Strengthening alliance between referral centres and proton centres may positively influence patient recruitment, and enhance opportunities for extensive and/or targeted clinical and translational research. Although a wide range of sample sizes was observed across the study designs of prospective studies, only one phase III study had > 200 patients, and ‘small’ sample size was cited as a limitation in prospective studies. Adequate sample size, statistical power and optimally designed studies are needed to ensure that the key research question(s) are addressed. The substantial increased capital cost, workforce requirements and the need for high quality evidence to make the case for health care system and insurer funding, requires high-level prospective evidence including randomised trials. This review suggests the need for substantial improvement in study design funding and delivery of future trials.

In addition, this review shows a particularly disappointing and significant concern; the paucity of patient reported outcome data as well the growing need for resources to capture late toxicity data beyond the primary analysis stage of the study. There is also the need for improved standard reporting of study design and outcomes in order to enable reproducibility, repeatability and comparability across similar patient cohorts.

### 4.1 Randomised trials in PBT

Only 9% (8/89) of the prospective studies included in this review randomised patients and of these RCTs, only one study compared protons with standard photon therapy. This finding resonates with the results of previous clinical reviews regarding the lack of high-level clinical evidence for PBT in several indications [53], [54] and further highlights the need for more randomised trials comparing PBT with standard photon therapy in adult patients. In the randomised phase II trial [36] of non-small cell lung cancer, no significant differences were observed in toxicity rates between passively scattered proton therapy (PSPT) and IMRT. Several possible reasons were suggested for the study’s findings [22] and these have raised further discussions and debate about the clinical advantages of PBT in the treatment of NSCLC. Although the trial used passively scattered proton beams, whether the use of a more advanced treatment planning technology such as pencil beam scanning or IMPT would have produced different results is unknown. This raises the issue of optimised old technology versus sub-optimal new therapy. The comparative effect of PBT to standard photons using meaningful endpoints is of crucial interest to stakeholders including patients, clinicians and financiers of PBT centres, particularly with advances in other radiotherapy modalities such as IMRT and stereotactic body radiation therapy (SBRT) and with the high cost of PBT [55], [56].

There is expert consensus that randomised proton trials in paediatrics would be unethical as the benefits are substantially greater allowing reduction in the risk of secondary cancers [57] and radiation effects on tissue growth and functional development [15], [58], [59]. Where it may be impractical to conduct randomised trials, well-designed prospective cohort studies may provide valuable insight in quantifying the advantage and developing strategies to mitigate or limit the disadvantages of PBT as patients can be monitored and any adverse events/reactions can be managed as they occur [59].

The number of retrospective studies appears to have increased over time and 19% (25/130) of authors of retrospective studies recommended the need for prospective analyses. Although the analysis of retrospective data may appear attractive due to the theoretical ready availability of data, without the additional cost of, and delays in, setting up a study or the barriers to recruiting patients prospectively, there are, however, methodological and technical disadvantages. These include access to complete data, differences in pre-treatment assessments, changes in treatment planning techniques over time, advances in treatment delivery technology (such as from passively scattered to pencil beam scanning), differences in treatment time-periods, and the heterogeneity of patient populations.

More prospective studies, especially well designed robust clinical trials in PBT are needed. The UK Proton Beam Clinical Trial Strategy Group has outlined an eight–point framework to support the development and delivery of high-quality clinical trials [60]. Statistical and clinical considerations on RCT design should include restriction of patient selection bias due to tumour characteristics, physician judgement and patient preference. When designing phase II studies, efficient strategies to utilize evidence gained in phase II and transition to definitive phase III evaluation should be developed.

Less than 50% (19/43) of the interventional studies reported a primary endpoint and specifically, just 13/21 phase II trials and 2/3 phase III. In selecting a primary endpoint consideration should be given to the level of evidence required to influence clinical practice. Of the primary endpoints reported, the majority were toxicity related although definitions, including assessment periods were variably reported, making replication challenging. No studies had PROMs as a primary or co-primary endpoint. Failure to define the primary endpoint/objective of a study makes evaluation of its success/failure prone to subjectivity and bias.

### 4.2 Collaboration between treatment centres

The majority of phase II and III trials were conducted in single PBT centres and small sample size was cited as a limitation in studies included in this review. Increased collaboration between PBT centres and referring photon centres would increase access to treatment and enrolment in trials. This may result in a more representative study sample, increased variability and power, which consequently make the findings applicable to the broader underlying population. Increased national and international collaboration during the planning and design stages of proton trials would further facilitate and strengthen the combination of results from several studies in a systematic review and/or *meta*-analysis as appropriate. Other challenges reported by authors include the high cost of setting up proton treatment facilities, which limits its availability, and the logistical considerations involved in patients travelling long distances to receive treatment. In the clinical trial setting, the impact of these will need to be considered in the design and conduct of the study. Although evidence suggests that patients are willing to travel to receive treatment [27], issues with cost coverage, support systems for patients when receiving treatment far away from home and the temporary absence of the familiar care team will need to be addressed.

### 4.3 Patient reported outcomes

Patient reported outcomes are progressively becoming a focal point in evaluating the effects of new interventions in health research, thus increasing awareness about the importance of obtaining patients’ perspectives during treatment and follow [61]. PROMS are assessed using QoL questionnaire scales and/or interviews in order to obtain a comprehensive evaluation of the patient’s QoL. Only 21% (19/89) of prospective studies in this review reported inclusion of PROMs as either a primary or secondary endpoint. For PBT, assessing QoL as a hypothesised benefit would provide information on the patient’s experience which consequently influences clinical decision-making in providing a holistic management protocol and improving the quality of patient care during and after treatment.

### 4.4 Increase in length of follow up

More than 30% (34/89) of authors have made recommendations for increased follow up duration to enable assessment of long term and late toxicities. This raises several questions about how to implement this in clinical practice: (a) what increase in length of follow up will significantly affect patient management and QoL from the clinician’s and patient’s perspectives?, (b) how will the collection and analyses of these data, such as PROMs using questionnaires, be performed? Notably, the answers to these will depend on several factors including the standard management protocol for the tumour site, characteristics of the patient cohort, funding platforms, human resources and accessing the required infrastructure. Given that one of the key predicted benefits of PBT is its effect on patient QoL in the short and long term, it would be important to have strategic deliberations on how long term follow up of patients may be achieved.

### 4.5 Radiotherapy quality assurance

Quality assurance procedures for radiotherapy clinical trials is recommended as this ensures alignment of specifications and enhances protocol adherence across participating sites, which consequently ensures reliability of trial results [62], [63]. The international collaboration of radiotherapy trials quality assurance (RTQA) groups around the world (Global Clinical Trial RTQA Harmonization Group) is well established and it is expected that PBT trials will employ these services during trial development and conduct.

### 4.6 Reporting of methods and findings

Comprehensive reporting of methods and findings are essential in order to ensure comparability between studies and enable evidence synthesis in systematic reviews and *meta*-analyses. The clinical and statistical considerations used in the design of the trial should be fully reported as this provides information on the robustness of the methodology and allows replication. Approximately 20% (17/78) of the included studies did not state the criteria used in the assessment of toxicities and more than 60% (50/75) did not state reporting timelines. Toxicity assessment is considered an important outcome in assessing the benefits of PBT, and several criteria are used in grading the observed clinician and patient reported adverse events. It is important that these are reported as the findings of a study informs clinical practice in decision making, patient management protocols during follow up and may influence policies on a broader scale. Standardised reporting of outcomes of interventional and observational studies in radiotherapy studies is needed. To address this, we suggest the development of a core or minimum outcome data set for PBT studies and/or the extension of the CONSORT statement [64] for use in radiotherapy RCTs.

We have combined phase I, feasibility and pilot studies for the purpose of this review as these design terms are used inconsistently in the literature. The framework on reporting of pilot and feasibility recommends clarification of the design with inclusion of these in the titles and/or abstracts [65].

Although our literature search has been thorough, it is not exhaustive and there were limitations in conducting this review. We did not include studies that were not published in the English language due to limited resources and in this way may have missed further eligible studies. In addition, we excluded published abstracts for which there were no corresponding full texts such as conference proceedings in order to ensure that all included articles were assessed on an equal basis. The treated tumour sites are not discussed individually as this was not within the scope of this review.

We have provided an overview of the study designs used in assessing the benefits of PBT in the treatment of various disease sites for adult and/or paediatric cohorts at PBT centres located across the world. Our findings highlight some of the challenges and limitations encountered in conducting these studies as well as the level of collaboration between PBT centres and other referring treatment centres. Furthermore, we have demonstrated the need for patient reported outcomes and long-term toxicity assessments in the evaluation of PBT.

With the development and inception of two NHS proton centres in the UK, and given the country’s record of accomplishment in conducting internationally practice-changing trials [66], there is an obligation to answer the question as to whether the dosimetric benefits of PBT translate into meaningful clinical benefits [67]. To maximise this opportunity, trials evaluating the effects of PBT must be of the highest quality, and should be designed in conjunction with experts in methodology, with reference to the points raised above. The challenge to the international radiotherapy research community is to deliver a broad portfolio of clinical trials that answer both clinical and translational research questions. This must include, wherever feasible, randomised designs to provide the highest level evidence possible. This will be achieved through a combination of studies in individual countries and through international participation.

## 5 Conclusions

Our findings clearly show there is limited randomised evidence for PBT and hence there is a need for further randomised trials to allow a robust assessment of the benefits of PBT. The development and inception of further PBT services across the globe provides an opportunity for well-designed prospective studies, including RCTs, and we recommend that researchers seek methodological input and support from clinical trial units in the design, development and throughout the course of the trial.

Infrastructure and resources to facilitate collection of long-term outcome data including QoL and late toxicities in cancer patients treated with PBT is recommended.

## Funding

This work has been funded by Cancer Research UK Centres Network Accelerator Award Grant (A21993) to the ART-NET consortium. Cancer Research UK had no involvement in the conduct of the study and decision to submit the article for submission.
