# Supplementary material for: Systematic review of methodology used in clinical studies evaluating the benefits of proton beam therapy
Source: Clin Transl Radiat Oncol. 2019 Jul 12;19:17–26. doi: 10.1016/j.ctro.2019.07.002 (PMC6660607; doi:10.1016/j.ctro.2019.07.002)
Supplement: Supplementary data 1 [file mmc1.docx]

**Supplementary Appendix**

**A.1 Search strategy used in each database**

- **Pubmed (with humans limit)**

(((((((((((((((((((((((((((((((((((((((proton beam therapy) OR proton beam radiation therapy) OR proton beam head neck) OR charged particle radiotherapy) OR charged-particle therapy) OR charged particle therapy) OR proton beam therapy breast) OR proton beam therapy head neck) OR proton beam therapy lung) OR proton beam therapy prostate) OR proton beam therapy brain) OR proton radiation therapy) OR proton beam treatment) OR proton beam therapy urology) OR proton bladder)) OR proton beam therapy paediatrics) OR proton beam therapy pediatrics) OR proton beam therapy children) OR proton radiotherapy) OR carbon-ion radiotherapy) OR intensity modified proton therapy)) OR proton radiation) OR intensity modulated proton therapy) OR charged particle radiotherapy) OR proton beam therapy oropharyn*) OR proton beam therapy nasopharyn*) OR proton beam therapy larynx) OR proton beam therapy hypopharyn*) OR proton beam therapy bladder))))) AND (((cancer) OR oncology) OR carcinoma))) AND ((((((((((randomised clinical trial) OR randomized clinical trial) OR randomised controlled trial) OR randomized controlled trial) OR comparative study)) OR quasi-randomised controlled trial) OR quasi-randomized controlled trial) OR quasi randomised clinical trials) OR quasi-randomized clinical trial)) NOT (((pump inhibitor) OR proton pump inhibitor) OR helicobacter)) AND Humans[Mesh]

- **Embase (Ovidsp)**


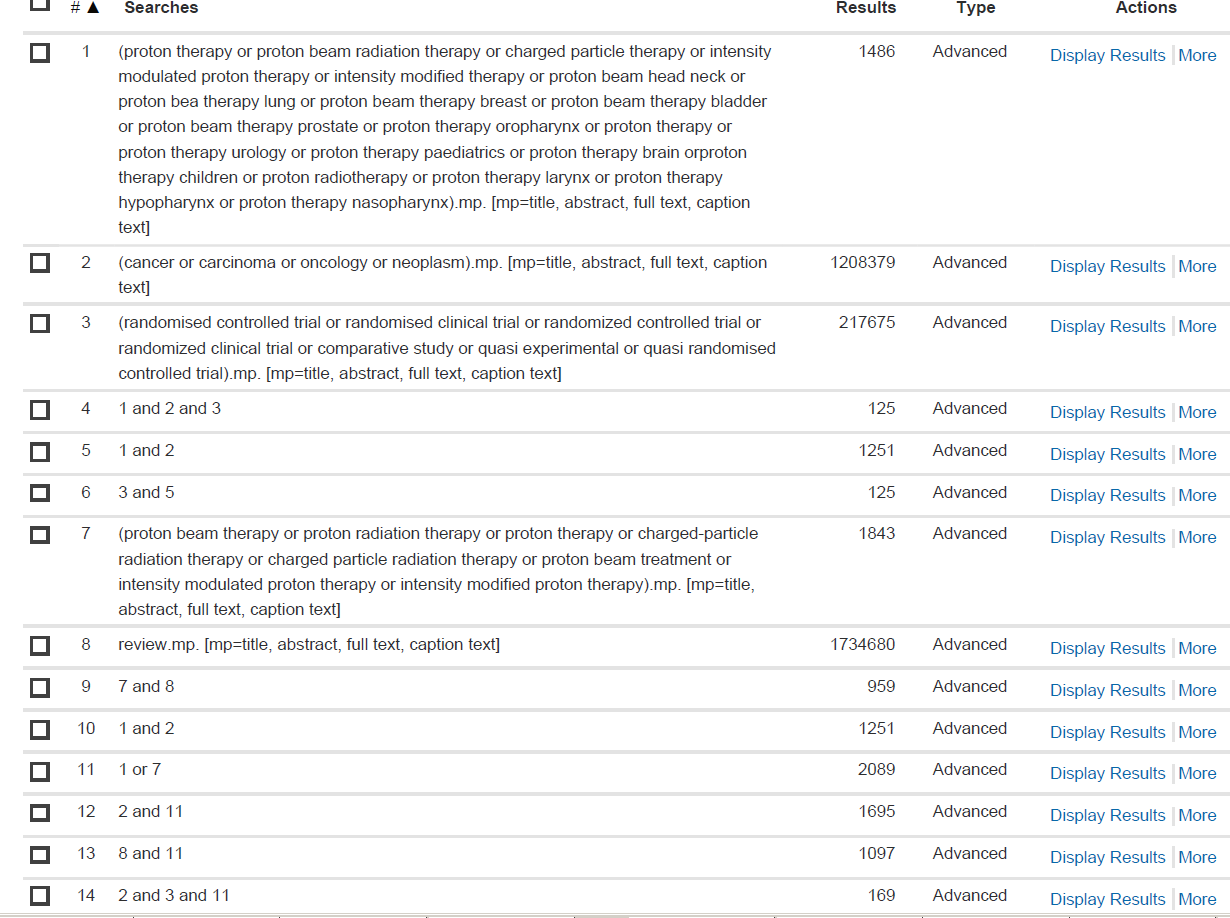


- **Cochrane database**


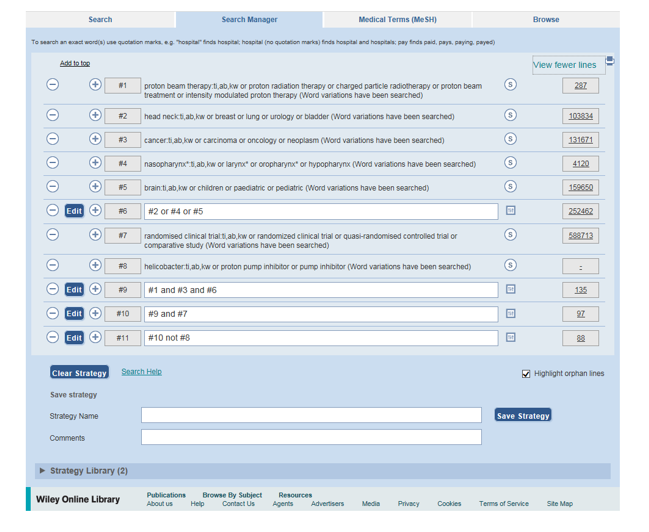


**A.2 Data extraction**

**Table A.1 Data extraction template for PBT Systematic Review**

| **List of data extracted from included articles** |
| --- |
| Author(s) |
| Year of publication |
| Title |
| Journal |
| Tumour site |
| Anatomical region |
| Cancer type |
| Stage of disease |
| Aim |
| Intervention(s) |
| Proton/radiotherapy dose details |
| Fractionation schedule |
| Full article or protocol or abstract |
| Gender |
| Age range of participants (years) |
| Location/Treatment centre(s) |
| Country |
| Sample size |
| Sample size details/comments |
| International study(yes/no) |
| Phase of study |
| Other design |
| Statistical design |
| Comparative or non-comparative |
| Number of treatment arms |
| Comparators |
| Randomised or non-randomised |
| Allocation ratio (if comparative/randomised study) |
| Retrospective or prospective |
| Source of patient data (if retrospective) (e.g. medical records, registry) |
| Single centre, multicentre or registry |
| Number of centres if multicentre |
| Blinding (randomised trials) |
| If blinding (single or double) |
| Informed consent obtained |
| Eligibility criteria stated |
| Intention to treat analysis |
| Treatment duration |
| Recruitment/treatment period (month year) |
| Length of recruitment (months) |
| Follow up time points |
| Length of follow up (months) |
| Statistical methods |
| Details of radiotherapy/PBT methods reported |
| Interim analyses (yes/no) |
| Completion rate (n/N)/Percentage drop out |
| Number included in primary analysis |
| Commented on missing data |
| Missing data reported |
| Missing data methods |
| Sensitivity analyses (yes/no) |
| Purpose of sensitivity analyses stated (yes/no) |
| Outcomes reported |
| PROMs reported |
| Proms questionnaire |
| PROMS method of collection |
| PROMS compliance rate |
| PROMS completion rate |
| Acute toxicities reported (yes/no) |
| Late toxicities reported (yes/no) |
| Grading system for toxicities |
| Definition of acute toxicities(timeline) |
| Definition of late toxicities(timeline) |
| Adverse events/reactions (if reported separately) |
| Commented on need for longer follow up (yes/no) |
| Reported on need to study late toxicities |
| Commented on limitations of retrospective analysis |
| Commented on need for comparative studies |
| Did comparative study favour proton (yes/no/not applicable) |
| Comments |

Abbreviations: PBT = Proton beam therapy; PROMs = Patient reported outcomes

**A.3**

**Table A.2 Details of proton beam therapy studies included in this review**

| Author, year | Condition | Aim | Study design | Treatment | Age range (years) | Sample size | Outcomes |
| --- | --- | --- | --- | --- | --- | --- | --- |
| Amsbaugh, 2012 [1] | Spinal ependymomas | To report acute toxicities and preliminary outcomes for paediatric patients with ependymomas of the spine treated with PBT. | Prospective; phase I/Feasibility/Pilot | PBT | 10 - 16 | 8 | Overall survival and progression free survival |
| Ares, 2009 [2] | Chordomas and chondrosarcoma | To evaluate effectiveness and safety of spot-scanning-based proton radiotherapy in skull base chordomas. | Prospective; phase I/II, non-randomised | PBT | 12 - 74 | 64 | Local tumour control, disease specific survival, overall survival and rates of toxicities. |
| Arimoto, 1991 [3] | Uterine cervical carcinoma | To evaluate the potential of proton beam radiotherapy as an alternative to conventional brachytherapy. | Phase I/Feasibility/Pilot | PBT | 58 - 70 | 15 | Local tumour control, survival and complications |
| Aziz, 2009 [4] | Uveal melanoma | To evaluate proton-beam radiotherapy in the management of uveal melanoma. | Retrospective | PBT | 29 - 88 | 76 | Visual acuity, incidence of toxicities, local tumour control, tumour dimensions, ocular complications and rate of enucleation. |
| Bensoussan, 2016 [5] | Choroidal melanoma | To evaluate proton beam therapy as a means to preserve the eye and spare some vision while not deteriorating survival in patients with large choroidal melanoma. | Retrospective | PBT | 19 - 89 | 492 | Local tumour control, overall survival and ocular complications. |
| Bhattasali, 2016 [6] | Adenoid cystic carcinoma | This article reports our institutional experience with PBT and cisplatin for unresectable head and neck adenoid cystic carcinoma. | Retrospective | PBT | Not stated | 9 | Local tumour control, acute and late toxicities. |
| Blanchard, 2016 [7] | Oropharynx cancer | To compare the clinical outcomes of IMRT and IMPT in a case matched analysis. | Prospective with retrospective (IMRT) control arm | PBT versus photons | 34 - 84 | 150 | Overall survival, progression free survival, toxicity assessment, emergency room visit and unscheduled hospitalization. |
| Boker, 2018 [8] | Uveal melanoma | Compared local tumour recurrence rates and metastasis rates between patients treated with adjuvant ruthenium brachytherapy and neoadjuvant PBT. | Retrospective | PBT with surgery | Not stated | 242 | Local tumour control, tumour recurrence rates, tumour-specific survival, rate of metastasis, visual acuity, enucleation rate, incidence of neovascular glaucoma and rate of iris rubeosis. |
| Bonnet, 2001 [9] | Non-small cell lung cancer | To evaluate the effects of irradiating lung cancer with protons or photons combined with protons on pulmonary function. | Prospective; phase I/II, non-randomised | PBT alone or combined with photons | Not stated | 25 | Pulmonary function: forced vital capacity, forced expiratory volume in one second, total lung capacity, inspiratory capacity, residual volume, and diffusing capacity |
| Boskos, 2009 [10] | Intracranial atypical and malignant meningioma | To evaluate the efficacy of conformal fractionated radiotherapy combining proton and photons beams after primary surgery for treatment of atypical and malignant meningioma. | Retrospective | PBT combined with photons | 11 - 72 | 24 | Local control and overall survival. |
| Brodin, 2012 [11] | Medulloblastoma | To compare the life years lost in paediatric medulloblastoma patients, between three radiotherapy treatment modalities; 3D-CRT, VMAT and IMPT. | Retrospective | PBT versus photons | 4 - 15 | 10 | Life years lost and late toxicity assessment |
| Brown, 2013 [12] | Medulloblastoma | To assess efficacy and toxicity of PBT in craniospinal irradiation compared with conventional photons for adults with medulloblastoma. | Retrospective | PBT | 16 - 49 | 40 | Overall survival, progression free survival, nausea/vomiting, dermatitis, haemoglobin level, white blood cells level, platelet count, and weight loss. |
| Bush, 2014 [13] | Breast carcinoma | To assess the efficacy and toxicity of PBT for partial breast irradiation. | Prospective; update of a phase II study | PBT | 41 - 83 | 100 | Disease free survival, recurrence free survival, overall survival, acute skin toxicity, late skin reactions, clinical fat necrosis, cases of rib fractures, clinical pneumonitis and cardiac events. |
| Bush, 2011 [14] | Hepatocellular carcinoma | To evaluate the safety and efficacy of PBT for hepatocellular carcinoma | Prospective; Phase II, non-randomised | PBT | 40 - 83 | 76 | Overall survival |
| Bush, 1999 [15] | Lung cancer | To assess the efficacy and toxicity of conformal proton beam radiotherapy for early-stage, medically inoperable non-small cell lung cancer. | Prospective; phase II, non-randomised | PBT alone or combined with photons | 54 - 87 | 37 | Toxicity assessment, overall survival, disease-free survival, local tumour control, regional tumour control, and rates of metastatic disease. |
| Bush, 2011 [16] | Breast cancer | To determine safety and efficacy of proton beam irradiation to deliver partial breast radiotherapy after lumpectomy for early stage breast cancer. | Prospective; phase II, non-randomised | PBT | 41 - 83 | 50 | Overall survival, disease free survival, cosmesis, acute and late toxicity assessment. |
| Bush, 2016 [17] | Hepatocellular carcinoma | To report interim analyses of PBT versus transarterial chemo-embolization. | Prospective; phase II, randomised | PBT versus transarterial chemoembolization | Not stated | 69 | Progression free survival, overall survival, local disease control, transplant outcomes, and toxicity assessment and length of hospitalisation after treatment. |
| Bush, 2004 [18] | Non-small cell-lung lung cancer | To determine the efficacy and toxicity of high-dose hypofractionated proton beam therapy for patients with clinical stage I lung cancer. | Prospective; phase II, non-randomised | PBT | 52 - 87 | 68 | Overall survival, disease specific survival rate, metastatic relapse rate, and local tumour control. |
| Caujolle, 2013 [19] | Uveal melanoma | To study the prognosis of different types of uveal melanoma recurrences treated by PBT. | Retrospective | PBT | 14.7 - 85 | 1102 | Overall survival, disease specific survival and local control. |
| Chang, 2013 [20] | Breast cancer | Effect of proton beam accelerated partial breast irradiation in patients with breast cancer. | Prospective; phase II, non-randomised | PBT | 40 - 69 | 30 | Treatment related toxicities, quality of life, percentage breast retraction assessment between the treated and untreated breast, and qualitative physician cosmetic assessment. |
| Chang, 2011a [21] | Non-small cell-lung lung cancer | To assess toxicity, failure patterns and survival in patients with unresectable non-small cell lung cancer after treatment with PBT and concurrent chemotherapy. | Prospective; phase II, non-randomised | PBT with concurrent chemotherapy | Not stated | 44 | Overall survival, progression free survival, recurrence rates, local control, and volume of lung exposed to PBT. |
| Chang, 2011b [22] | Non–small cell lung cancer | To analyse the toxicity and patterns of failure of proton therapy given in ablative doses for medically inoperable early-stage non–small cell lung cancer. | Prospective; phase I/II, non-randomised | PBT | 61 - 83 | 18 | Overall survival, disease free survival and local control. |
| Char, 2002 [23] | Uveal melanoma | To evaluate late (>5 years) radiation failures after uveal melanoma treatment with radiotherapy. | Retrospective | Charged particle (PBT or helium ions) or Iodine 125 brachytherapy | Not stated | 996 | Local recurrence rates. |
| Char, 2003 [24] | Uveal melanoma | To assess effect of proton with laser induced hyperthermia on decreasing exudative retinal detachments. | Retrospective | PBT combined with laser-induced hyperthermia | Not stated | 56 | Visual acuity, days to resorption and sub retinal fluid resorption. |
| Chen, 2013 [25] | Spine and sacral chordoma | To report the results of high dose proton based definitive radiotherapy for unresected spinal chordomas. | Retrospective | PBT alone or combined with photons | Not stated | 24 | Overall survival, progression free survival, distant metastasis free survival, local control, acute and late toxicity assessment. |
| Chiba, 2005 [26] | Hepatocellular carcinoma | To report results of patients with hepatocellular carcinoma treated with proton beam therapy. | Retrospective | PBT alone or combined with transarterial embolization and percutaneous ethanol injection. | 41 - 84 | 165 | Local tumour control, overall survival, performance status, histopathologic changes, vascular invasion, incidence of adverse reactions and rate of recurrence. |
| Childs, 2012 [27] | Parameningeal rhabdomyosarcoma | To report the clinical outcome and late side effect profile of proton radiotherapy in the treatment of children with parameningeal rhabdomyosarcoma. | Retrospective | PBT | 0.4 - 17.7 | 17 | Late side effects, recurrence rates, failure free survival and overall survival. |
| Christopherson, 2014 [28] | Sinonasal carcinoma | To evaluate the long-term effectiveness of radiotherapy in the treatment of sinonasal undifferentiated carcinoma. | Retrospective | PBT alone or combined with photons, post-chemotherapy | 23 - 83 | 23 | Rates of local control, regional control, local-regional control, distant metastasis-free survival, cause specific survival, and overall survival |
| Ciernik, 2011 [29] | Osteosarcoma | To assess clinical outcome and the role of PBT for local control of osteosarcoma in all patients who received PBT or mixed photons-proton radiotherapy. | Retrospective | PBT alone or combined with photons, post-chemotherapy | 2 - 76 | 55 | Local control, disease-free survival, overall survival, and long term toxicity, |
| Coen, 2012a [30] | Prostate cancer | To report long-term quality of life outcome for men treated with conformal protons. | Prospective | PBT | 49 - 78 | 95 | Urinary function (incontinence, obstruction/irritation) bowel problems and sexual function. |
| Coen, 2012b [31] | Prostate cancer | Case-matched analysis comparing high-dose external-beam radiation for prostate cancer delivered on Proton Radiation Oncology Group 95-09, a randomized trial, with permanent prostate brachytherapy over the same era in patients with low to intermediate risk prostate cancer. | Retrospective | Photons with PBT boost (EBRT) versus brachytherapy | 47 - 76 | 282 | *Biochemical failure (using the Phoenix definition), overall survival and freedom from distant metastasis. |
| Coen, 2011 [32] | Prostate cancer | To test the safety and efficacy of 82 GyE delivered with conformal proton radiation in men treated for localized prostate cancer. | Prospective; phase II, non-randomised | PBT | 44 - 80 | 84 | Acute and late toxicities of the genito-urinary and gastrointestinal tracts. |
| Colaco, 2013 [33] | Small cell lung cancer | To report outcomes for the first known series of limited-stage SCLC patients treated with proton therapy and a dosimetric comparison of lung and oesophageal doses with IMRT. | Retrospective | PBT | 52 - 76 | 6 | Overall survival, progression free survival and recurrence free survival. |
| Courdi, 1999 [34] | Uveal melanoma | To present first results of patients treated for uveal melanoma. | Retrospective | PBT | 10 - 88 | 538 | Cause specific survival rate, local control, eye retention rate, rate of distant metastasis, visual outcome, tumour height and size. |
| Cuaron, 2015 [35] | Breast cancer | To report on early toxicity in breast cancer patients treated with post-operative PBT. | Retrospective | PBT | 29 - 86 | 30 | Acute toxicities |
| Damato, 2005 [36] | Choroidal melanoma | To determine ocular outcomes and survival after PBT of choroidal melanoma. | Prospective | PBT | 19 - 85 | 349 | Visual acuity, local treatment failure rate and mortality rate. |
| Damato, 2005 [37] | Iris melanoma | To evaluate the early outcomes of PBT of iris melanoma, in terms of visual acuity and ocular complications. | Retrospective | PBT | 21 - 76 | 88 | Corrected visual acuity, presence of recurrent /persistent tumour growth, ocular complications (cataract, glaucoma), enucleation rate and time to cataract diagnosis. |
| DeLaney, 2014 [38] | Spine sarcomas | To evaluate effect of high dose photons/proton for spine sarcomas. | Prospective; phase II, non-randomised | PBT combined with photons | Not stated | 50 | Local control, recurrence free survival and overall survival. |
| Demizu, 2014 [39] | Head and neck cancers | To retrospectively analyse treatment outcomes after particle therapy using PBT or carbon ions for mucosal melanoma of the head and neck, as well as to compare PBT and carbon ion therapy. | Retrospective | PBT or carbon ions | 33 - 89 | 62 | Overall survival, progression free survival, local control and toxicity assessment |
| Dendale, 2006 [40] | Uveal melanoma | To report the results of PBT in patients with uveal melanoma. | Retrospective | PBT | 15 - 90 | 1406 | Local control, overall survival, metastasis-free survival, ocular complications, rates of enucleation and visual acuity. |
| Deraniyagala, 2014 [41] | Skull base chordoma | To report on the experience of using PBT for skull base chordomas. | Retrospective | PBT | Not stated | 33 | Local control and overall survival. |
| Desjardins, 2012 [42] | Uveal melanoma | To report results of comparing a series of patients treated with PBT with data published in the literature. | Retrospective | PBT | 14 - 95 | 2413 | Overall survival, rate of metastasis, local control, secondary enucleation, thickness of the tumour scar and ocular toxicities (retinal detachment, glaucoma, cataract, optic neuropathy, radiation maculopathy, vitreous haemorrhage) and visual acuity. |
| Desjardins, 2006 [43] | Uveal melanoma | To determine whether systematic transpupillary thermotherapy after proton beam radiotherapy could have a beneficial effect. | Prospective; phase III, randomised | PBT alone versus PBT with transpupillary thermotherapy | 22 - 88 | 151 | Ocular functional assessment (visual acuity, presence or absence of palpebral sequelae, keratitis, cataract, glaucoma, retinal detachment, optic neuropathy or maculopathy, and inflammation), thickness of the tumour scar, presence of local recurrence, rates of metastasis and secondary enucleation. |
| Duttenhaver, 1983 [44] | Prostate cancer | To retrospectively compare outcomes in patients with prostate cancer treated with combined x-ray therapy plus proton therapy with patients treated by x-ray therapy alone. | Retrospective | PBT versus photons | 46 - 85 | 180 | Overall survival, progression free survival, disease free survival and recurrence free survival. |
| Eaton, 2016 [45] | Medulloblastoma | To compare long-term disease control and overall survival between children treated with proton and photons radiation therapy for standard-risk medulloblastoma. | Retrospective | Photons alone versus photons with PBT boost | 3.3 - 21.9 | 88 | Overall survival, recurrence free survival and patterns of failure. |
| Egger, 2001 [46] | Uveal melanoma | To report local tumour control and survival after PBT of uveal melanoma. | Retrospective | PBT | 9 - 89 | 2435 | Local tumour control rates, recurrence rate, tumour size, and overall survival. |
| Egger, 1997 [47] | Uveal melanoma | To report results of proton beam therapy in patients treated for unilateral uveal melanoma. | Retrospective | PBT | Not stated | 1351 | Overall survival, tumour control, eye retention probability, and rates of ocular complications (neovascular glaucoma, radiation induced optic neuropathy and atrophy). |
| Fang, 2015 [48] | Prostate cancer | To assess whether PBT for prostate cancer was associated with differing toxicity compared with IMRT. | Retrospective | PBT versus photons | Not stated | 394 | Overall survival, acute and late toxicities. |
| Feuvret, 2016 [49] | Chondrosarcoma | To assess the effect of the quality of surgery and radiation therapy parameters on local control and overall survival in patients with skull base tumours treated with protons alone or protons and photons. | Retrospective | PBT alone versus PBT with photons boost | 12 - 83 | 159 | Local control, overall survival, early and late side effects. |
| Fitzek, 1999 [50] | Glioblastoma multiforme | To assess whether dose escalation to 90 CGE with conformal proton/photons in accelerated fractionation would improve local tumour control and patient survival. | Prospective; phase II, non-randomised | PBT combined with photons | 21 - 68 | 23 | Treatment tolerance (3 months after radiation), treatment toxicity (>3 months after treatment), overall survival and quality of survival. |
| Fitzek, 2002 [51] | Olfactory neuroblastoma and Neuroendocirne carcinoma | To report the results of patients who were diagnosed with olfactory neuroblastoma or neuroendocirne carcinoma whose primary management included chemotherapy and proton beam therapy. | Prospective | PBT combined with photons | 26 - 67 | 19 | Overall survival, metastasis free survival, local failure rate, toxicity assessment and rate of salvage therapy. |
| Frank, 2014 [52] | Squamous cell carcinoma and adenoid cystic carcinoma | To report the first clinical experience and toxicity of multi-field optimization IMPT for patients with head and neck tumours. | Prospective | PBT | Not stated | 15 | Toxicity assessment and tumour response (classified as complete or partial). |
| Fuji, 2014 [53] | Sinonasal mucosal malignant melanoma | To evaluate the role of high-dose PBT in patients with sinonasal mucosal melanoma. | Retrospective | PBT | 55 - 81 | 20 | Acute and late toxicity assessment, rate of tumour recurrence, overall survival, progression free survival, local control rate, and rate of distant metastasis. |
| Fujii, 2013 [54] | Non-small cell lung cancer | To compare the clinical outcomes and late toxicities of proton therapy with carbon ion therapy. | Retrospective | PBT or carbon ions | 39 - 89 | 111 | Overall survival, progression free survival, local control rates and late toxicities. |
| Fujii, 2015 [55] | Non-small cell lung cancer | To report the treatment outcomes of particle therapy for indeterminate pulmonary nodules diagnosed as stage I non-small cell lung cancer, and a comparative analysis involving pathologically proven lung cancer. | Retrospective | PBT or carbon ions | 52 - 87 | 165 | Overall survival, progression-free survival, local control, distant progression-free survival rates, and toxicity assessment. |
| Fukumitsu, 2012 [56] | Nasal cavity and paranasal sinus carcinoma | To investigate the clinical features, prognostic factors, and toxicity of treatment for unresectable carcinomas of the nasal cavity and paranasal sinus treated with PBT. | Retrospective | PBT alone vs PBT with photons boost | 30 - 83 | 17 | Overall survival, local recurrence rate, metastasis and toxicity assessment. |
| Fukumitsu, 2009 [57] | Hepatocellular carcinoma | To evaluate the efficacy and safety of hypofractionated PBT for patients with hepatocellular carcinoma. | Prospective | PBT | Not stated | 51 | Overall survival, cause specific free survival, local control, local progression, treatment side effects and serum alpha-fetoprotein level. |
| Fuss, 2001 [58] | Choroidal melanoma | To evaluate the efficacy and safety of PBT for medium- and large-size choroidal melanoma with focus on preservation of the eye and its function. | Retrospective | PBT | 26 - 89 | 78 | Local control, metastasis free survival rate, overall survival, disease-specific survival, and enucleation rate. |
| Galland-Girodet, 2014 [59] | Breast cancer | To present long-term outcomes and toxicities of a prospective feasibility trial using either protons or 3-dimensional conformal photons-based accelerated partial-breast irradiation techniques. | Prospective; phase I/II, non-randomised | PBT VS photons | 60 - 63 | 98 | Local control, survival, assessment of toxicities, cosmetic outcomes, physician assessment for fat necrosis. |
| Gardner, 2002 [60] | Prostate cancer | To determine the long-term normal tissue effects of combined photons and conformal protons delivered to the prostate in patients with locally advanced prostate cancer. | Retrospective | photons with PBT boost | 50 - 81 | 39 | Late toxicity assessment - genitourinary and gastrointestinal. |
| Gragoudas, 2002 [61] | Uveal melanoma | To report the long term results after treatment with proton beam therapy in patients with intraocular melanoma. | Retrospective | PBT | 11 - 93 | 2069 | Local recurrence rate, tumour related mortality, enucleation, and vision loss. |
| Gragoudas, 2000 [62] | Choroidal melanoma | To determine the effect of a reduction in PBT dose from standard 70 CGE to 50 CGE on treatment outcomes. | Prospective; phase III, randomised | PBT | 19 - 86 | 188 | Local recurrence rate, metastasis rate, vision loss, ocular complications, incidence of visual field defects, increase in defects and tumour control. |
| Grant, 2015 [63] | Paediatric salivary gland tumours | To evaluate toxicity profiles for paediatric patients with parotid or submandibular tumours treated with adjuvant conventional radiotherapy. | Retrospective | PBT | 6 - 18 | 24 | Acute toxicity assessment |
| Gray, 2013 [64] | Prostate cancer | To review prospectively reported PROM data in patients with localised prostate cancer treated with 3D-CRT, IMRT or PBT. | Retrospective | PBT VS photons | 47 - 83 | 371 | Patient reported quality of life score on the bowel/rectal, urinary irritation/obstruction, and urinary incontinence domains of PCSI and EPIC questionnaires. |
| Greenberger, 2014 [65] | Paediatric low-grade Gliomas | To report outcomes of 32 paediatric patients treated with PBT. | Retrospective | PBT alone or PBT with photons boost | 2.7 - 21.5 | 32 | Progression free survival, overall survival, incidence of endocrinopathy, and neurocognitive outcomes (Full scale IQ, Verbal Comprehension Index, Perceptual Reasoning Index). |
| Gunn, 2016 [66] | Oropharyngeal squamous carcinoma | To assess disease control and toxicity of proton therapy for patients with head and neck cancer. | Prospective; phase II, non-randomised | PBT | 37 - 84 | 50 | Progression free survival, overall survival, disease control, toxicity, functional outcomes, and patterns of failure. |
| Habl, 2016 [67] | Prostate cancer | To explore the safety and feasibility of primary hypofractionated irradiation of the prostate with protons and carbon ions in a raster scan technique. | Prospective; phase II, randomised | PBT or carbon ions | 40 - 80 | 92 | Toxicity assessment, PSA progression free survival, overall survival and quality of life. |
| Habrand, 2008 [68] | Paediatric skull base and cervical canal low-grade bone malignancies | To evaluate outcomes and tolerance of high-dose photons and proton therapy in the management of skull base and cervical canal primary bony malignancies in children. | Retrospective | PBT alone or combined with photons | 6 - 17 | 30 | Local control, toxicity assessment, overall survival, progression free survival. |
| Hashimoto, 2006 [69] | Hepatocellular carcinoma | To retrospectively evaluate the safety and effectiveness of repeated proton beam therapy for newly developed or recurrent hepatocellular carcinoma. | Retrospective | PBT | 40 - 89 | 27 | Local control, acute and late toxicity assessment. |
| Hata, 2007 [70] | Stage I non–small-cell lung cancer | To present treatment outcomes of hypofractionated high-dose proton beam therapy for stage I non–small-cell lung cancer. | Prospective; phase I/II, non-randomised | PBT | 51 - 85 | 21 | Acute and late toxicity assessment, actuarial survival, local progression free survival and disease free rates. |
| Hata, 2006 [71] | Hepatocellular carcinoma | To report the treatment results from proton beam therapy in hepatocellular carcinoma patients with limited treatment options because of coexisting diseases and unfavourable conditions. | Retrospective | PBT | 47 - 76 | 21 | Acute and late Toxicity assessment, Actuarial survival, local progression free survival, disease free survival rates. |
| Hata, 2006 [72] | Hepatocellular carcinoma | To report on outcomes for HCC patients with severe cirrhosis treated with proton beam therapy. | Retrospective | PBT | 51 - 69 | 19 | Tumour control, failure patterns , disease free survival, overall survival, acute and late toxicity assessment |
| Hata, 2005 [73] | Hepatocellular carcinoma | To report the results of proton beam therapy in the treatment of patients who had HCC with portal vein tumour thrombus. | Retrospective | PBT | 42 - 80 | 12 | Overall survival, progression free survival, local tumour control, failure patterns, acute and late toxicity assessments. |
| Hata, 2007 [74] | Hepatocellular carcinoma | To investigate the safety and efficacy of proton beam therapy for aged patients with HCC | Retrospective | PBT | 80 - 85 | 21 | Acute and late toxicity assessment, local tumour control and pattern failure, cause-specific and disease free survival rates. |
| Hattangadi, 2012 [75] | Neuroblastoma | To report the early outcomes for children with high-risk neuroblastoma treated with PBT and to compare the dose distributions for IMRT, 3D-CPT, and IMPT to the postoperative tumour bed | Retrospective | PBT | 0.8 - 4 | 9 | Local tumour control, rate of metastasis, acute and late toxicity assessment. |
| Hauswald, 2012 [76] | Glioma | To assess feasibility and toxicity of PBT in patients with low-grade glioma | Retrospective | PBT | Not stated | 19 | Local tumour control and toxicity assessment. |
| Herr, 2014 [77] | Esthesioneuroblastoma | To reassess treatment outcomes and complications in patients with esthesioneuroblastoma treated with craniofacial resection followed by PBT with or without chemotherapy. | Retrospective | PBT alone or with chemotherapy | 11 - 77 | 22 | Overall survival, disease free survival, local/regional/distant control rates and side effects. |
| Higgins, 2017 [78] | Non-small cell lung cancer | To analyse outcomes and predictors associated with proton radiation therapy for non-small cell lung cancer | Retrospective | PBT versus photons | 18 - 90 | 243822 | Overall survival |
| Holliday, 2015 [79] | Nasopharyngeal cancer | to determine if patients treated with IMPT had significantly less acute and chronic morbidity during radiation therapy when compared to case-matched controls treated with IMRT | Retrospective | PBT | 18 - 55 | 30 | Gastrotomy tube placement, toxicity assessment, local control and survival, weight loss and incidence of swallowing dysfunction. |
| Holliday, 2015 [80] | Chordomas and chrondosarcomas | To evaluate patients treated with PBT for chordoma and chondrosarcomas post-surgery, to report local control , relapse free and overall survival | Retrospective | PBT alone or PBT with photons boost | 10 - 82 | 19 | Local tumour control, relapse free and overall survival, pain level, Karnofsky performance status, and weakness affecting function. |
| Hoppe, 2012 [81] | Prostate cancer | To evaluate patient reported health related quality of life after PBT for prostate cancer | Prospective | PBT | 41 - 60 | 262 | Mean EPIC scores (sexual summary, sexual function, and sexual bother scores), incidence of potency or erectile dysfunction score on the IIEF scale. |
| Hoppe, 2014 [82] | Prostate cancer | To compare patient-reported QOL outcomes after proton therapy PBT and IMRT and for prostate cancer. | Prospective; PBT group compared with retrospective IMRT control group | PBT | 40 - 89 | 1447 | EPIC composite question - bowel, urinary, or sexual function |
| Hug, 1999 [83] | Chordomas and chrondosarcomas | To assess treatment efficacy in patients who received PBT for skull base chordomas and chrondosarmas | Prospective | PBT | 10 - 85 | 58 | Local tumour control, patient survival and treatment failure rates. |
| Hug, 2002 [84] | Paediatric low-grade astrocytomas | To evaluate safety and efficacy of PBT for intra-cranial astrocytomas | Retrospective | PBT | 2 - 18 | 27 | Toxicities, local-recurrence free survival and overall survival |
| Hug, 2002 [85] | Paediatric skull base tumours (mesenchymal neoplasms) | To review a group of 29 paediatric patients with mesenchymal base of skull tumours, all treated with PBT. | Retrospective | PBT | 1 - 19 | 29 | Disease specific survival, overall survival, patterns of failure, local tumour control, and treatment related side effects. |
| Hungerford, 1997 [86] | Ocular melanoma | To identify predictive factors for rubeotic glaucoma after PBT. | Retrospective | PBT | 19 - 83 | 127 | Rubeosis free ocular survival |
| Igaki, 2004 [87] | Skull base chordoma | To evaluate clinical results of PBT for patients with skull base chordoma. | Retrospective | PBT alone or PBT with photons boost | 14 - 74 | 13 | Local tumour control, locoregional recurrence, cause-specific survival, disease free survival and overall survival. |
| Ishikawa, 2015 [88] | Oesophageal cancer | to evaluate the outcomes of PBT concurrently combined with chemotherapy consisting of cisplatin and 5-fluorouracil for oesophageal cancer | Prospective | PBT with concurrent chemotherapy | 52 - 79 | 40 | Overall survival, cause-specific survival and locoregional control, treatment related toxicity assessments. |
| Iwata, 2018 [89] | Prostate cancer | To report on survey of the long-term outcomes of PBT for prostate cancer. | Retrospective | PBT | 62 - 73 | 1291 | Biochemical relapse-free survival, overall survival, cause-specific survival, biochemical relapse-free rates, clinical relapse-free rates; and incidence of grade ≥2 late gastrointestinal and genito-urinary toxicities. |
| Iwata, 2010 [90] | Non–small-cell lung cancer | To analyse the safety and efficacy of high-dose proton therapy and carbon-ion therapy applied to stage I non-small cell lung cancer. | Prospective | PBT or carbon ions | 48 - 89 | 80 | Overall survival, cause-specific survival, local- control, and disease-free survival rates, incidence of metastasis and toxicity assessment. |
| Iwata, 2013 [91] | Non–small-cell lung cancer | To evaluate the clinical outcome of particle therapy for T2a/bN0M0 non-small cell lung cancer. | Prospective | PBT or carbon ions | 57 - 92 | 70 | Overall survival, local control, progression free survival, rate of metastasis, regional recurrence rates and toxicity assessment. |
| Jimenez, 2013 [92] | Pediatric medulloblastoma  and supratentorial primitive neuroectodermal tumours | To report the early outcomes for very young children with medulloblastoma or supratentorial primitive neuroectodermal tumour treated with upfront chemotherapy followed by 3-dimensional proton radiation therapy. | Retrospective | PBT | 23 - 55 | 15 | Local failure rate, overall survival, toxicity assessment, changes in vertical height, cognitive and adaptive functioning. |
| Kagei, 2003 [93] | Carcinoma of the uterine cervix | To determine the efficacy of PBT in patients with carcinoma of the uterine cervix and compare results to those achieved by conventional radiotherapy. | Retrospective | PBT | 36 - 77 | 25 | Overall survival, local control, cause-specific survival, acute and late toxicity. |
| Kamran, 2014 [94] | Uveal melanoma | To report results in patients with uveal metastasis treated with PBT. | Retrospective | PBT | Not stated | 77 | Overall survival, time to tumour progression, Local failure, incidence of uveal metastasis, post treatment adverse effects, retinal detachment resolution and visual acuity after treatment. |
| Kanemoto, 2014 [95] | Non-small cell lung cancer | To determine the disease control rate and prognostic factors associated with recurrence of centrally and peripherally located stage I non-small cell lung cancer treated using high-dose PBT. | Retrospective | PBT | 51 - 86 | 74 | Overall survival, disease-specific survival, progression free survival, local control rates, recurrence rates, acute and late toxicity assessments, |
| Kawashima, 2005 [96] | Hepatocellular carcinoma | To evaluate the safety and efficacy of proton beam radiotherapy (PRT) for hepatocellular carcinoma. | Prospective; phase II, non-randomised | PBT | 48 - 87 | 30 | Tumour control, overall survival, progression free survival, recurrence rate, disease free survival, and adverse events assessment. |
| Kil, 2013 [97] | Prostate cancer | To investigate post-treatment changes in serum testosterone in low- and intermediate-risk prostate cancer patients treated with hypofractionated passively scattered proton radiotherapy. | Prospective | PBT | 41 - 83 | 217 | Serum testosterone levels |
| Kim, 2010 [98] | Parapapillary melanoma | To evaluate parapapillary choroidal melanoma | Retrospective | PBT | 14 - 81 | 93 | Vision loss, rates of papillopathy, spontaneous vision recovery, vision retention rate and vision improvement. |
| Kim, 2013 [99] | Prostate cancer | To investigate the feasibility of hypofractionated PBT in treatment of prostate cancer. | Prospective; phase II, randomised | PBT | 44 - 85 | 82 | Acute and late toxicity assessments,  quality of life, biochemical control and biochemical failure free survival. |
| Komatsu, 2011 [100] | Hepatocellular carcinoma | To evaluate the clinical outcome of proton and carbon ion therapy for hepatocellular carcinoma | Prospective | PBT or carbon ions | 19 - 83 | 343 | Overall survival, local control, acute and late toxicity assessment. |
| Konstantinidis, 2015 [101] | Choroidal melanoma | To evaluate the effect of transpalpebral PBT of uveal melanoma, in terms of ocular morbidity and local tumour control. | Retrospective | PBT | Not stated | 63 | Local tumour control, ocular retention rate, and rate of madarosis. |
| Koyama, 2003 [102] | Esophageal carcinoma | To investigate the efficacy and toxicity associated with the use of PBT for radical radiation therapy in oesophageal carcinoma. | Prospective | PBT alone or combined with photons | 45 - 95 | 30 | Overall survival, disease specific survival, local recurrence rates, and acute toxicity assessment. |
| Kozak, 2006 [103] | Breast cancer | To present the initial clinical results with proton, 3D conformal, external beam partial-breast irradiation. | Prospective; phase I/II, non-randomised | PBT | 46 - 75 | 20 | Recurrent disease, cosmetic outcome, toxicity, and patient satisfaction. |
| Kuhlthau, 2012 [104] | Brain cancer | To describe the health-related quality of life of a cohort of children with brain tumours treated with proton radiotherapy. | Prospective | PBT | 2 - 18 | 142 | Health related quality of life using the Paediatric Quality of Life inventory. |
| Ladra, 2014 [105] | Rhabdomyosarcoma | To assess disease control and to describe acute and late adverse effects of treatment with proton radiotherapy in children with rhabdomyosarcoma | Prospective; phase II, non-randomised | PBT | 0.6 - 19.5 | 57 | Disease control rate, acute and late toxicity assessment, event-free survival, overall survival, and local control rate. |
| Lane, 2015 [106] | Uveal melanoma | To determine the long-term risk of dying in patients treated for uveal melanoma using proton beam therapy. | Retrospective | PBT | 10.3 - 94.2 | 3088 | Disease specific mortality, disease specific hazard rates and cumulative all-cause mortality rates. |
| Lane, 2011 [107] | Peripapillary and parapapillary tumours | To examine ocular outcomes and survival after proton irradiation in patients with peripapillary and parapapillary melanomas | Retrospective | PBT | 14 - 91 | 573 | Melanoma-related mortality, tumour recurrence, vision loss, enucleation and ocular complications. |
| Leiser, 2016 [108] | Rhabdomyosarcoma | To assess clinical outcomes in children with rhabdomyosarcoma treated with pencil beam scanning proton therapy. | Retrospective | PBT | 0.8 - 15.5 | 83 | Quality of life score using Paediatric Quality of Life inventory, toxicity assessment, local control rate, and overall survival. |
| Lewis, 2016 [109] | Nasopharyngeal carcinoma | to present recurrence rates, survival outcomes, and toxicities experienced by the first 9 patients treated with IMPT for Nasopharyngeal carcinoma | Retrospective | PBT with concurrent chemotherapy | 17 - 59 | 9 | Acute and late toxicity assessment, local and regional control rates, distant metastatic free survival rate, overall survival, feeding tube utilization and weight loss. |
| Liao, 2018 [110] | Non–small-cell lung cancer | To compare outcomes of passive scattering proton therapy versus IMRT, both with concurrent chemotherapy, for inoperable non–small-cell lung cancer. | Prospective; phase II, randomised | PBT versus photons | 33 - 85 | 149 | Incidence of severe (grade 3) radiation pneumonitis or local failure and overall survival. |
| Lin, 2016 [111] | Gynaecologic cancer | To report the acute toxicities associated with pencil beam scanning PBT for whole pelvis radiation therapy in women with gynaecologic cancers | Prospective | PBT alone or with concurrent chemotherapy | 23 - 76 | 11 | Acute toxicity assessment |
| Lin, 2012 [112] | Squamous cell carcinoma, adenocarcinoma and mixed adeno and neuroendocrine | To report on toxicities and clinical outcomes when treating patients with PBT and concurrent chemotherapy | Retrospective | PBT with concurrent chemotherapy | 35 - 86 | 62 | Acute toxicity assessment, pathologic complete response rate, complete response rate, local regional recurrences, distant metastatic-free survival and overall survival. |
| Linton, 2015 [113] | Adenoid cystic carcinoma (salivary gland) | To report outcomes of proton therapy in head and neck adenoid cystic carcinoma. | Retrospective | PBT | 21 - 73 | 26 | Local control rate, distant metastasis rate, overall survival, acute and late toxicity assessment. |
| Lucas, 2015 [114] | Esthesioneuroblastoma | To report clinical outcomes and toxicity from our institutional experience utilizing proton therapy in the paediatric and adolescent population for the treatment of esthesioneuroblastoma. | Retrospective | PBT with concurrent chemotherapy | 6 - 21 | 8 | Overall survival, locoregional control rate, metastasis rate, acute and late toxicity assessment, |
| Lumbroso-Le Rouic, 2006 [115] | Iris melanoma | To describe the results in terms of local control, eye preservation and systemic evolution of iris melanomas treated by PBT irradiation. | Retrospective | PBT | 17 - 82 | 21 | Overall survival, local recurrence rate, local tumour response rate, incidence of ocular complications and rate of secondary enucleation. |
| Macdonald, 2011 [116] | Uveal melanoma | To review the outcome of Scottish patients undergoing treatment for uveal melanoma with PBT. | Retrospective | PBT | 27.7 - 89.8 | 147 | Eye retention rate, metastatic disease rate, disease-specific survival and radiation complications. |
| MacDonald, 2013a [117] | Breast cancer | To report acute toxicities and feasibility of proton delivery for 12 women treated with post mastectomy proton radiation with or without reconstruction. | Prospective; phase I/Feasibility/Pilot | PBT | 31 - 68 | 12 | Incidence of skin toxicity, acute radiation pneumonitis, and fatigue. |
| MacDonald, 2013b [118] | Ependymoma | To report outcomes for patients treated with PBT for intracranial ependymoma. | Retrospective | PBT | 0.25 - 20 | 70 | Progression free survival, overall survival, patterns of failure, toxicity assessment, local control, distant control, neurocognitive assessment, endocrine and auditory outcomes. |
| Makita, 2014 [119] | Cholangiocarcinoma | To retrospectively evaluate the efficacy and toxicity of proton beam therapy for the treatment of unresectable and inoperable cholangiocarcinoma. | Retrospective | PBT | 41 - 84 | 28 | Overall survival, progression-free survival and local control |
| Maquilan, 2014 [120] | Gliomas and meningiomas | Severity and frequency of acute toxicities of PBT in patients with low-grade gliomas and meningiomas. | Prospective | PBT | Not stated | 23 | Fatigue, anorexia, nausea, vomiting, headache and insomnia |
| Marucci, 2011 [121] | Uveal melanoma | To retrospectively compare survival in recurrent uveal melanoma, between patients treated by enucleation or by a second course of fractionated PBT. | Retrospective | PBT versus enucleation | 25 - 80 | 73 | Overall survival and rate of metastasis |
| Matsuzaki, 1994 [122] | Hepatocellular carcinoma | To report results of proton therapy in treatment of patients with hepatocellular carcinoma. | Prospective; phase I/Feasibility/Pilot | PBT alone or with concurrent chemotherapy | Not stated | 24 | Mortality rate, tumour size, local tumour control, pathological changes (presence of viable cancer cells), measurable serum alpha-fetoprotein levels, rate of adverse events, quality of life and toxicity assessment. |
| McAvoy, 2013 [123] | Non-small cell lung cancer | To report 5 year outcomes on the efficacy and toxicity of PBT for reirradiation of patients with non-small cell lung cancer. | Retrospective | PBT | 42 - 85 | 33 | Overall survival, progression-free survival, locoregional control rate, and distant metastasis-free survival. |
| McDonald, 2016 [124] | Nasopharynx and paranasal sinus cancers | To evaluate acute toxicity endpoints in a cohort of patients receiving head and neck radiation with PBT or IMRT. | Retrospective | PBT | 22 - 77 | 40 | Acute toxicity assessment |
| McDonald, 2013 [125] | Chordoma | To report the results in patients reirradiated with proton therapy for recurrent or progressive chordoma, with or without salvage surgery. | Prospective | PBT | 26 - 78 | 16 | Overall survival, toxicity assessment, disease-specific survival, metastasis rate and local control rate. |
| McGee, 2013 [126] | Prostate cancer | Retrospectively evaluate the toxicity profiles of prostate cancer patients with large prostates (>60cm3) treated with definitive PBT at a single institution. | Retrospective | PBT | Not stated | 186 | Biochemical failure, genitourinary toxicity and rate of gastrointestinal toxicity. |
| McGovern, 2014 [127] | Atypical teratoid/rhabdoid tumour | To evaluate a single institution experience in the use of proton radiation for the treatment of paediatric atypical teratoid/rhabdoid tumour of the CNS | Retrospective | PBT | 4 - 55 | 31 | Overall survival and progression-free survival |
| Mendenhall, 2012 [128] | Prostate cancer | To report early outcomes with image-guided proton therapy for prostate cancer. | Prospective; phase I/Feasibility/Pilot | PBT | 40 - 88 | 211 | Overall survival, progression-free survival, PROMs (IPSS, EPIC, IEFF questionnaires), PSA response, and toxicity assessment |
| Mendenhall, 2014 [129] | Prostate cancer | To report five-year outcomes with image-guided proton therapy for prostate cancer. | Prospective | PBT | 40 - 88 | 211 | Patterns of disease progression, PSA response, overall survival, progression-free survival, patient reported outcomes (IPSS and EPIC questionnaires), and toxicity assessment |
| Mizumoto, 2010 [130] | Esophageal cancer | Retrospective evaluation of the efficacy and safety of high-dose PBT | Retrospective | PBT alone or combined with photons | 47 - 95 | 51 | Toxicity assessment, overall survival, local control and pattern of recurrence. |
| Mizumoto, 2010 [131] | Glioblastoma mulitforme | Evaluate the safety and efficacy of hyperfractionated concomitant boost proton radiotherapy. | Prospective; phase I/II, non-randomised | PBT boost with chemotherapy | 31 - 76 | 21 | Overall survival and MRI-change free survival |
| Moeller, 2011 [132] | Medulloblatoma | To determine whether proton radiotherapy techniques spares early ototoxicity for children with medulloblastoma. | Prospective | PBT | 3 - 16 | 23 | Hearing sensitivity, mean post-radiation thresholds and rate of ototoxicity. |
| Morimoto, 2014 [133] | Head and neck cancers with skull base invasion | To determine the oncological outcomes and complications of patients with unresectable primary head and neck cancers invading the skull base. | Retrospective | PBT or carbon ions | 24 - 81 | 57 | Toxicity, survival and local progression free survival |
| Mosci, 2009 [134] | Uveal melanoma | To evaluate the results of treatment with PBT in patients with intraocular melanoma. | Prospective | PBT | Not stated | 368 | Local tumour control, eye retention rate and survival |
| Mouw, 2014 [135] | Retinoblastoma | To report long-term outcomes of using PBT to treat retinoblastoma patients. | Retrospective | PBT | 0.08 - 2.5 | 49 | Enucleation-free survival, tumour control, ocular complications, visual outcomes |
| Naeser, 1998 [136] | Uveal melanoma | To describe the early and late clinical changes following PBT for patients with uveal melanoma after 5 years follow--up. | Prospective | PBT | 31 - 73 | 20 | Eye retention rate, enucleation rate, tumour recurrence rate, and mortality rate. |
| Nakamura, 2017 [137] | Olfactory neuroblastoma | To clarify the efficacy and feasibility of PBT for olfactory neuroblastoma. | Retrospective | PBT | 20 - 87 | 42 | Overall survival, progression-free survival, the site of the first progression, and adverse effects |
| Nakayama, 2011 [138] | Non–small-cell lung cancer | To assess the effect of proton beam therapy in the treatment of patients with stage II or III non-small cell lung cancer who were inoperable or ineligible for chemotherapy because of co-existing disease or refusal. | Retrospective | PBT | 51 - 83 | 35 | Overall survival, progression-free survival and local progression free survival |
| Nakayama, 2010 [139] | Non-small-cell lung cancer | To confirm results of safety and effectiveness of treating medically inoperable stage I non-small cell lung cancer patients with PBT. | Retrospective | PBT | 52 - 86 | 55 | Progression free survival, local control, overall survival and rate of adverse events |
| Nakayama, 2009 [140] | Hepatocellular carcinoma | To present the survival and prognostic data from patients treated with proton beam therapy for hepatocellular carcinoma. | Retrospective | PBT | 47.4 - 85.4 | 333 | Toxicity assessment and overall survival |
| Nguyen, 2015 [141] | Non-small cell lung cancer | To assess outcomes for patients with locally advanced non-small cell lung cancer prospectively treated with concurrent proton therapy and chemotherapy. | Prospective | PBT with concurrent chemotherapy | 28 - 95 | 134 | Overall survival, disease free survival, local recurrence and rate of distant metastasis. |
| Nichols, 2013 [142] | Pancreatic cancer | To review treatment toxicity for patients with pancreatic and ampullary cancer treated with proton therapy. | Prospective | PBT with concurrent chemotherapy | Not stated | 22 | Toxicity assessment, weight loss, local and regional tumour control, distant metastasis, and overall survival. |
| Nichols, 2012 [143] | Prostate cancer | To determine whether testosterone suppression is associated with proton radiotherapy in the treatment of prostate cancer. | Prospective | PBT | 41 - 86.4 | 150 | Testosterone level |
| Nihei, 2005 [144] | Prostate cancer | To assess the feasibility and safety of high-dose radiotherapy for prostate cancer using proton boost therapy following photons radiotherapy. | Prospective; phase II, non-randomised | photons with PBT boost | 54 - 87 | 30 | Acute and late toxicity assessment and PSA-failure free survival |
| Nihei, 2006 [145] | Non-small cell lung cancer | To evaluate safety and efficacy of high-dose proton beam therapy for stage I non–small cell lung cancer. | Retrospective | PBT | 63 - 87 | 37 | Acute and late toxicity assessment, local response rate, local progression-free survival, disease progression-free survival and overall survival |
| Nihei, 2011 [146] | Prostate cancer | To estimate prospectively the incidence of late rectal toxicities after PBT for organ-confined prostate cancer. | Prospective; phase II, non-randomised | PBT | 51 - 82 | 151 | Acute and late toxicity assessment disease specific survival, and biochemical relapse free survival, |
| Nishimura, 2007 [147] | Olfactory neuroblastoma | To examine the utility of PBT for olfactory neuroblastoma. | Retrospective | PBT | 30 - 84 | 14 | Overall survival, local progression free survival and progression free survival |
| Noel, 2003 [148] | Chordoma and Chondrosarcoma | To report on outcomes in patients treated with fractionated photons and proton radiation for chordoma or chondrosarcoma of the base of the skull and the cervical spine. | Prospective | PBT combined with photons | 14 - 85 | 67 | Overall survival, local tumour control, rate of relapse, acute and late toxicities |
| Noël, 2005 [149] | Chordoma | To define the prognostic factor for local control and overall survival among 100 consecutive patients with chordoma of the base of skull or upper cervical spine treated by fractionated irradiation combining proton and photons beams. | Retrospective | PBT combined with photons | 8 - 85 | 103 | Dose delivered, overall survival, local control and adverse event rates. |
| Ohkawa, 2015 [150] | Intrahepatic cholangiocarcinoma | To evaluate the outcome of proton beam therapy for patients with unresectable intrahepatic cholangiocarcinoma. | Retrospective | PBT | 82 - 82 | 20 | Local tumour control, overall survival, acute and late toxicity assessment |
| Okano, 2012 [151] | Nasal and sinonasal malignancies | To evaluate the efficacy and feasibility of induction chemotherapy using docetaxel, cisplatin and S-1 followed by proton beam therapy concurrent with cisplatin. | Retrospective | PBT with concurrent chemotherapy | 28 - 60 | 13 | Progression-free survival, overall survival, toxicity assessment, local recurrence rate and complete response rate. |
| Patel, 2016 [152] | Choroidal melanoma | To review visual outcomes in patients undergoing irradiation with PBT for tumours located within 1 disc diameter of the fovea. | Retrospective | PBT | 14 - 91 | 351 | Rates of vision retention of 20/40 or better, radiation induced ocular complications and visual loss |
| Phan, 2016 [153] | Head and neck cancers (various) | To report on the clinical outcomes and toxicity with the use of PRT for head and neck reirradiation. | Retrospective | PBT | 29 - 80 | 60 | Locoregional control, locoregional failure-free survival, overall survival, progression-free survival, distant metastasis-free survival, acute and late toxicity assessment. |
| Pommier, 2006 [154] | Skull base  adenoid cystic carcinoma | To determine the treatment outcome and prognostic factors in patients with adenoid cystic carcinoma of the skull base treated with PBT. | Retrospective | PBT | 25 - 66 | 23 | Locoregional control, disease-free survival, overall survival rate, rate of distant metastasis, ocular complications, toxicity assessment, neurologic and endocrine outcomes. |
| Pugh, 2016 [155] | Prostate cancer | To report prostate cancer outcomes, toxicity, and quality of life in men treated with proton beam therapy. | Prospective | PBT | 45 - 82 | 423 | Overall survival, disease specific survival, biochemical control, and patterns of failure, toxicity assessment, and quality of life scores. |
| Rahmi, 2014 [156] | Iris melanoma | To report the clinical features and outcomes of iris melanomas treated by proton beam therapy. | Retrospective | PBT | 22 - 82 | 36 | Local tumour control, rate of metastasis and ocular complications |
| Remick, 2017 [157] | Non-small cell lung cancer | To report the first institutional experience with proton therapy for postoperative radiation therapy in patients with non-small cell lung cancer and assess early toxicities and outcomes. | Retrospective | PBT versus photons | 38 - 80 | 61 | Overall survival, progression free survival and toxicity assessment |
| Rich, 1985 [158] | Chordoma | To review results of treatment with PBT combined with photons in patients diagnosed with chordoma. | Retrospective | PBT alone or combined with photons | Not stated | 48 | Overall survival and rate of tumour regression |
| Riechardt, 2014 [159] | Uveal melanoma | To evaluate survival and ocular outcomes in recurrent uveal melanoma treated with PBT as salvage therapy. | Retrospective | PBT, brachytherapy, transpupillary thermotherapy, laser photocoagulation, CyberKnife radiation, or photodynamic therapy | 32 - 84 | 48 | Local tumour control, visual acuity, metastasis free survival and overall survival |
| Rombi, 2012 [160] | Ewing's sarcoma | To report the preliminary clinical outcomes including late effects on paediatric Ewing’s sarcoma patients treated with PBT. | Retrospective | PBT | 1.8 - 21 | 30 | Overall survival, event free survival, disease-specific survival, local control, acute and late toxicity assessment. |
| Rombi, 2013 [161] | Paediatric chordoma and chondrosarcoma | To evaluate clinical results of fractionated spot-scanning proton radiation therapy. | Prospective | PBT | 3.7 - 20.8 | 26 | Local control, overall survival, acute and late toxicity assessment. |
| Romesser, 2016 [162] | Head and neck cancer (various) | To report the first multi-institutional clinical experience using curative-intent PBT for reirradiation in recurrent head and neck cancer. | Retrospective | PBT | Not stated | 92 | Overall survival, locoregional control, distant metastasis-free survival, acute and late toxicity assessment. |
| Rossi, 1999 [163] | Prostate cancer | To evaluate the toxicity and response to treatment with conformal PBT in patients with prostate cancer. | Prospective | PBT | Not stated | 643 | Biochemical disease free survival, PSA level, acute and late toxicity assessment. |
| Rotondo, 2015 [164] | Spine chordoma | To assess treatment outcomes and prognostic factors after high-dose PBT with or without surgery. | Retrospective | PBT | 5 - 88 | 126 | Toxicity assessment, local control, regional control, locoregional control, distant control, overall survival and neurological status. |
| Rundle, 2007 [165] | Iris melanoma | To report results of PBT for iris melanoma. | Retrospective | PBT | 11 - 61 | 15 | Eye retention rate and tumour control rate |
| Rwigema, 2017 [166] | Limited-stage small cell lung cancer | To evaluate clinical outcomes and toxicities in patients treated with PBT for limited-stage small cell lung cancer. | Prospective | PBT with concurrent chemotherapy | 57 - 81 | 30 | Local control rate, recurrence free survival, overall survival rates and toxicity assessment. |
| Sachsman, 2015 [167] | Non-Hodgkin's lymphoma | To evaluate the disease control, toxicities and radiation dose delivered to various organs at risk  using PBT with or without chemotherapy among a cohort of patients with non- Hodgkin lymphoma. | Prospective | PBT alone or with concurrent chemotherapy | 4 - 66 | 11 | Toxicity assessment and rate of disease progression |
| Sachsman, 2014 [168] | Pancreatic adenocarcinoma | To review early outcomes and assess toxicities for patients with unresectable pancreatic cancer. | Prospective; phase I/II, non-randomised | PBT with concurrent chemotherapy | 51 - 86 | 12 | Overall survival, progression-free survival, distant metastasis-free survival, freedom from local progression rates, toxicity assessment and weight loss. |
| Sandinha, 2014 [169] | Iris melanoma | To report on recurrence of iris melanoma after proton beam therapy. | Retrospective | PBT | Not stated | 150 | Local tumour control and ocular retention rate |
| Schlienger, 1996 [170] | Uveal melanoma | To present preliminary results of patients with uveal melanoma treated with protons. | Prospective | PBT | Not stated | 146 | Enucleation rate, toxicity assessment, overall survival and rate of metastasis |
| Schönfeld, 2014 [171] | Choroidal melanoma | To evaluate long-term outcomes of proton beam radiotherapy in the treatment of choroidal melanoma of the intermediate zone of the fundus. | Retrospective | PBT | Not stated | 62 | Tumour thickness, rate of distant metastasis, local tumour relapse rate, enucleation rate and visual acuity. |
| Seddon, 1985 [172] | Uveal melanoma | To compare survival rates between PBT and enucleation. | Retrospective | PBT versus enucleation | Not stated | 516 | Metastasis-free survival, disease related mortality rate and overall survival. |
| Seddon, 1990 [173] | Uveal melanoma | To compare survival in patients treated with PBT with those who had enucleation. | Retrospective | PBT versus enucleation | Not stated | 1051 | Overall survival and disease related mortality rate |
| Seibel, 2016 [174] | Uveal melanoma | To evaluate the incidence, risk factors, and dosages of PBT associated with cataract development, and long-term visual outcomes after treatment of uveal melanoma. | Retrospective | PBT | 16 - 72 | 258 | Visual acuity, ocular complications, rate of metastasis, and local recurrence rate. |
| Seibel, 2015 [175] | Uveal melanoma | To evaluate the risk factors, recurrence rates, retreatments, and long-term patient outcomes following PBT for uveal melanoma. | Retrospective | PBT | Not stated | 982 | Overall survival, metastasis-free survival, rate of metastasis, local recurrence rate, local tumour control rate and visual acuity. |
| Sejpal, 2011 [176] | Non-small cell lung cancer | To report early experience with acute and subacute toxicity from PBT and concurrent chemotherapy for locally advanced non-small cell lung cancer. | Phase I/II, non-randomised compared with retrospective data from photon studies | PBT versus photons | 38 - 82 | 202 | Toxicity assessment and overall survival |
| Sethi, 2014 [177] | Retinoblastoma | To compare the risk of second malignancy in patients with retinoblastoma who were treated with photons and PBT. | Retrospective | PBT versus photons | 0.2 - 11.7 | 86 | Rate of secondary malignancy, cumulative incidence of radiotherapy-induced or in-field malignancies , ocular complications, |
| Sherman, 2016 [178] | Low grade glioma | To understand neurocognitive effects of proton radiation therapy in patients with low-grade glioma. | Prospective | PBT | 22 - 56 | 20 | Overall survival, progression free survival change in neurocognitive function, neuroendocrine function and quality of life. |
| Shioyama, 2003 [179] | Non-small cell lung cancer, squamous cell carcinoma, adenocarcinoma and large-cell carcinoma. | Report on the efficacy of patients treated with PBT for non-small cell lung cancer | Prospective | PBT | 25 - 87 | 51 | Disease free survival, overall survival, cause-specific survival, local control rate, acute and late toxicity assessment. |
| Shipley, 1979 [180] | Prostate cancer | To treat the prostatic tumour volume to a dose 5% or 11% higher than conventional megavoltage x-ray | Prospective | Photons with PBT boost | Not stated | 17 | Toxicity assessment and local recurrence rate |
| Shipley, 1995 [181] | Prostate cancer | To evaluate the possible increased efficacy of a higher dose of radiation on the local recurrence rate and patient survival in men with locally advanced prostate cancer. | Prospective; phase III, randomised | Photons alone versus photons with PBT boost | 46 - 85 | 202 | Acute and late toxicity assessment, overall survival, disease-specific survival, local tumour control and recurrence free survival. |
| Sikuade, 2015 [182] | Choroidal melanoma | To present results of the use of stereotactic radiosurgery and PBT to treat posterior uveal melanoma over a 10 year period. | Retrospective | PBT versus stereotactic radiosurgery | 17 - 87 | 191 | Overall survival, local control rates, local recurrence rate, eye retention rate, ocular complications, visual acuity, visual loss, |
| Sio, 2016 [183] | Oropharyngeal cancer | To report PROMs in patients with oropharyngeal cancer treated with either chemotherapy and IMPT or chemotherapy and IMRT. | Retrospective | PBT versus photons | Not stated | 81 | Patient reported outcomes using MDASI-HN questionnaire |
| Slater, 1988 [184] | Chordoma or chondrosarcoma | To evaluate the endocrine function of patients who received PBT irradiation for tumours of the upper clivus. | Retrospective | PBT | 16 - 65 | 19 | Thyroid function, gonadotropin function, corticotrophin function and serum prolactin levels |
| Slater, 2004 [185] | Prostate cancer | To evaluate results of conformal PBT for localized prostate cancer. | Prospective | PBT alone or combined with photons | 44 - 90 | 1255 | Biochemical disease free survival, PSA levels and toxicity assessment |
| Slater, 2005 [186] | Oropharyngeal cancer | To assess accelerated fractionation using photons and proton radiation to improve local control and reduce complications in treating locally advanced oropharyngeal cancer. | Prospective; phase I/Feasibility/Pilot | Photons with PBT boost | Not stated | 29 | Locoregional control, rate of metastatic disease, disease free survival and toxicity assessment. |
| Song, 2014 [187] | Brain cancer | To compare the acute toxicity of craniospinal irradiation using proton beam relative to that of conventional photons beam in children with brain tumours. | Prospective | PBT versus photons | 3 - 18 | 43 | Acute toxicity assessment, serum thrombopoeitin levels, haematological profile and serum thrombopoietin levels. |
| Sugahara, 2009 [188] | Hepatocellular carcinoma | To evaluate the efficacy of PBT for patients presenting with portal vein tumour thrombosis in the setting of hepatocellular carcinoma. | Retrospective | PBT | Not stated | 35 | Progression-free survival, local progression-free survival, overall survival, acute and toxicity assessment |
| Sugahara, 2010 [189] | Hepatocellular carcinoma | To investigate the safety and efficacy of PBT in patients with large hepatocellular carcinoma. | Retrospective | PBT | 45 - 90 | 22 | Overall survival, local control rates, recurrence rates, progression-free survival, rate of metastasis and toxicity assessment. |
| Sugahara, 2005 [190] | Oesophageal cancer | To present the results of PBT for patients with oesophageal cancer. | Retrospective | PBT | 45 - 95 | 46 | Overall and progression-free survival, response rate, serum levels of alpha-fetoprotein or protein-induced by vitamin K absence or antagonists–II, acute and late toxicity assessment. |
| Suneja, 2013 [191] | CNS malignancies | To examine the acute toxicity for children with CNS malignancies treated with PBT. | Retrospective | PBT | Not stated | 48 | Acute toxicity assessment, Lansky performance status and weight loss. |
| Takaoka, 2017 [192] | Bladder cancer | To elucidate the oncological outcomes, prognostic factors and toxicities of proton beam therapy in trimodal bladder-preserving therapy for muscle-invasive bladder cancer. | Retrospective | PBT combined with photons and surgery | 36 - 85 | 70 | Overall survival, progression-free survival, time to progression, acute and late toxicity assessment |
| Talcott, 2010 [193] | Prostate cancer | To determine long-term, patient-reported, dose-related toxicity. | Prospective | PBT combined with photons | 45.2 - 79.5 | 393 | Rates of urinary incontinence, urinary obstruction and irritation, bowel problems, sexual dysfunction, and quality-of-life (using PCSI scale). |
| Terashima, 2012 [194] | Pancreatic cancer | To assess the feasibility and efficacy of gemcitabine-concurrent PBT for locally advanced pancreatic cancer. | Prospective; phase I/II, non-randomised | PBT with concurrent chemotherapy | 45 - 83 | 50 | Local control, distant metastasis, local progression-free survival, progression-free survival, overall survival and toxicity assessment. |
| Timmermann, 2007 [195] | Soft tissue sarcoma | To investigate the feasibility of spot-scanning PBT for soft tissue tumours in childhood. | Prospective; phase I/Feasibility/Pilot | PBT | 1.4 - 14.1 | 16 | Acute and late toxicity assessment, tumour response rate, local control rate, progression-free survival, overall survival and quality of life. |
| Tokuuye, 2004 [196] | Head and neck cancers | To evaluate the effectiveness and feasibility of PBT for head and neck cancers. | Retrospective | PBT | Not stated | 33 | Overall survival, progression-free survival, local control, acute and late toxicity assessment |
| Tran, 2012 [197] | Choroidal melanoma | To report on outcomes for patients with peripapillary choroidal melanoma treated with proton therapy. | Retrospective | PBT | 34 - 86 | 59 | Local control rate, overall survival, metastasis-free survival, enucleation rate, incidence of optic neuropathy, visual acuity and ocular complications. |
| Truong, 2009 [198] | Sphenoid sinus malignancies | To determine treatment outcome and prognostic factors in patients with locally advanced primary sphenoid sinus malignancy treated with PBT. | Retrospective | PBT or carbon ions | 17 - 78 | 20 | Disease-free survival, overall survival, distant metastasis rate, acute and late toxicity assessment, incidence of endocrine and neurological complications. |
| Tsina, 2005 [199] | Choroidal cancer | To describe the clinical outcomes of patients treated by proton beam irradiation for choroidal metastatic tumours. | Retrospective | PBT | 29 - 87 | 63 | Visual acuity, tumour response rate, local control rate and toxicity assessment. |
| Vargas, 2016 [200] | Prostate cancer | To evaluate changes in quality of life or adverse events among prostate cancer patients treated with hypofractionation. | Prospective | PBT | 52 - 75 | 49 | Toxicity assessment and quality of life using American Urological Association Symptom Index. |
| Vavvas, 2010 [201] | Uveal melanoma | To study the clinical profile and prognosis of young patients with uveal melanoma treated with PBT. | Retrospective | PBT | 20 - 20 | 68 | Overall survival |
| Viswanathan, 2011 [202] | Brain cancer | To characterize the rate of occurrence and type of pituitary hormone dysfunction in children with brain tumours who received proton irradiation and to compare the rates and type of dysfunction in this patient population with those in children with brain tumours who received both conventional and proton radiation therapy. | Retrospective | PBT alone or combined with photons | 3.6 - 17.4 | 31 | Rates of pituitary hormone dysfunction, pituitary deficiencies and endocrinopathies. |
| Weber, 2016 [203] | Chondrosarcoma of the skull base | To assess the long-term tumour chondrosarcoma patients treated with pencil beam scanning only PBT. | Prospective | PBT | 10.2 - 70 | 77 | Local tumour control, toxicity free survival, overall survival |
| Weber, 2006 [204] | Sinonasal malignacies | To investigate the visual outcomes of patients with advanced sinonasal malignancies treated with proton/photons accelerated fractionated radiation. | Retrospective | PBT combined with photons | Not stated | 36 | Acute and late visual/ocular toxicity assessment |
| Weber, 2005 [205] | Chondrosarcoma of the skull base | To assess the clinical results of spot scanning PBT in the treatment of skull base chordomas and low-grade chondrosarcomas. | Prospective | PBT | 15 - 77 | 29 | Progression free survival, overall survival and local tumour control rate |
| Weber, 2015 [206] | Atypical teratoid rhabdoid tumour of CNS | To assess the early clinical results of pencil beam scanning PBT in the treatment of young children with non-metastatic atypical teratoid/rhabdoid tumour of the CNS. | Prospective | PBT with concurrent chemotherapy | 0.4 - 2.3 | 15 | Overall survival, progression-free survival, late toxicity free survival, quality of life, and toxicity assessment. |
| Westover, 2012 [207] | Non-small cell lung cancer | To report the outcomes for the patients treated with proton SBRT. | Retrospective | Proton SBRT | Not stated | 15 | Overall survival, regional, distant, and local failure rates and toxicity assessment. |
| Willerding, 2016 [208] | Uveal melanoma | To describe results after neoadjuvant proton beam irradiation followed by transscleral resection of large uveal melanoma. | Retrospective | PBT with surgery | 81 - 81 | 17 | Rate of eye retention, local tumour control rate, metastasis-free survival, visual acuity, and toxicity assessment. |
| Wilson, 1999 [209] | Choroidal melanoma | To compare the efficacy of iodine-125 (125I) and ruthenium-106 (106Ru) episcleral plaque radiation therapy and PBT in the treatment of choroidal melanoma. | Retrospective | PBT versus episcleral plaque radiation | 13 - 87 | 597 | Loss of visual acuity, local recurrence rate, metastasis rate, ocular complications, enucleation rate, time to enucleation, and mortality rate |
| Yasuda, 2012 [210] | Chordomas of Skull base and cervical spine | To review the clinical outcome of patients with a chordoma at the skull base, cranio-cervical junction or cervical spine. | Prospective | PBT combined with photons | 11 - 68 | 30 | Progression-free survival, overall survival and rate of metastasis |
| Yock, 2010 [211] | Medulloblastoma | To describe the clinical outcomes of patients undergoing proton radiotherapy for medulloblastoma. | Prospective; phase II, non-randomised | PBT | 3.5 - 22 | 60 | Overall survival, progression-free survival, audiology, endocrine dysfunction and neurocognitive decline. |
| Yock, 2016 [212] | Medulloblastoma | To assess late complications, acute side-effects, and survival following treatment with PBT in children with medulloblastoma. | Prospective; phase II, non-randomised | PBT with concurrent chemotherapy | 3 - 21 | 59 | Overall survival, progression free survival, endocrine dysfunction and audiology assessment. |
| Yonemoto, 1997 [213] | Prostate cancer | To evaluate the toxicity and response of a proton boost combined with photons for the treatment of locally advanced prostatic carcinoma. | Prospective; phase I/II, non-randomised | PBT combined with photons | 54 - 81 | 106 | Acute and late toxicity assessment, PSA levels, local tumour control, rate of metastatic disease, and local recurrence. |
| Yu, 2013 [214] | Prostate cancer | To compare the patterns of PBT use, cost, and early toxicity with those of intensity-modulated radiotherapy in patients with prostate cancer. | Retrospective | PBT versus photons | Not stated | 27647 | Patterns of care, treatment costs and toxicity assessment of gastrointestinal and genito-urinary tracts. |
| Yuh, 2004 [215] | Medulloblastoma | To report results on first use of protons for treatment of medulloblasotma in children. | Prospective | PBT | 3 - 4 | 3 | Acute toxicity assessment |
| Zenda, 2015 [216] | Nasal cavity, para-nasal sinuses, or involving the skull base cancers | To clarify the late toxicity profile of PBT in patients with malignancies of the nasal cavity, para-nasal sinuses, or involving the skull base. | Retrospective | PBT | 17 - 84 | 90 | Late toxicity assessment |
| Zenda, 2011 [217] | Mucosal melanoma of the head and neck | To assess the clinical benefit of PBT for mucosal melanoma of the head and neck. | Retrospective | PBT | 56 - 79 | 11 | Overall survival, progression-free survival, rates of recurrence and distant-metastasis, acute and late toxicity assessment. |
| Zenda, 2011 [218] | Head and neck cancers | To clarify the clinical profile of PBT for unresectable malignancies of the nasal cavity and paranasal sinuses. | Retrospective | PBT | 22 - 84 | 39 | Overall survival, progression-free survival, local control rates, acute and late toxicity assessment |
| Zietman, 2010 [219] | Prostate cancer | To test the hypothesis that increasing radiation dose delivered to men with early-stage prostate cancer improves clinical outcomes. | Prospective; phase II, randomised | Photons with PBT boost | 45 - 91 | 393 | Local failure rate, biochemical failure rate, overall survival and toxicity assessment. |

Abbreviations: PBT = Proton beam therapy; IMPT = Intensive modulated proton therapy; IMRT = Intensive modulated radiation therapy; CRT= Conformal radiotherapy; 3D-CRT = Three dimensional conformal radiotherapy; 3D-PRT = Three dimensional proton radiotherapy; VMAT = Volumetric modulated arc therapy; PRT = Proton radiotherapy; XRT = X-ray radiotherapy; PROMs = Patient reported outcomes; CPT = Conformal proton therapy; NSCLC = Non-small cell lung cancer; CNS = Central nervous system; SBRT = Stereotactic body radiation therapy; PSA = Prostate specific antigen; PCSI = Prostate Cancer Symptom Indices ; EPIC = Expanded Prostate Cancer Index Composite; IIEF = International Index of Erectile Function; PedQoL = Paediatric Quality of Life; GyE = Gray equivalent CGE = Cobalt gray equivalent; ^╪^The Phoenix (nadir + 2) definition defines failure as any rise of >2 ng/mL above the lowest prior post treatment PSA level.

**Table A.3 Forms of interventions in prospective studies (n=89)**

|  | Interventions in prospective studies | Frequency |
| --- | --- | --- |
| Comparative | PBT (dose levels) | 3 |
|  | PBT vs photons | 6 |
|  | PBT vs carbon ions | 1 |
|  | PBT vs transarterial chemoembolization | 1 |
|  | PBT alone vs PBT with transpupillary thermotherapy | 1 |
|  | Photon alone vs photon with PBT boost | 2 |
|  | PBT combined with photons (PBT dose levels) | 1 |
|  |  |  |
| Non-comparative | PBT | 45 |
|  | PBT or carbon ions | 3 |
|  | PBT with concurrent chemotherapy | 9 |
|  | PBT alone or with concurrent chemotherapy | 3 |
|  | Photons with PBT boost | 4 |
|  | PBT combined with photons | 6 |
|  | PBT alone or combined with photons | 4 |

Abbreviations: PBT = Proton beam therapy

**A.4 Figures showing distribution of sample sizes of prospective studies included in review**

Figure A.1 Sample sizes of interventional studies (n=43)

Figure A.2 Sample sizes of observational studies (n=46)

**A.5 Questionnaires used to measure patient reported outcomes reported in studies included in this review.**

- - EORTC QLQ-C30
  - EORTC QLQ-PR25 (prostate cancer module)
  - EORTC QLQ BR23 (breast cancer module)
  - Expanded prostate cancer index composite (EPIC)
  - Prostate Cancer Symptom Indices (PCSI)
  - Karnofsky Performance scale
  - Lansky performance scale
  - American Urological Association (AUA ) score
  - Urinary quality of life score
  - Sexual dysfunction scale
  - Validated assessments of sexual bowel complications of treatment, and
  - PCSI quality of life-urinary
  - Internationals Prostate Symptom Score (IPSS)
  - International Erectile Function Form (IEFF 5)
  - Paediatric Quality of Life (PEDQoL)
  - Quality of life questionnaire for childhood cancer survivors (PEDQUOL)

**References**

[1]. Amsbaugh MJ, Grosshans DR, McAleer MF, et al. Proton therapy for spinal ependymomas: Planning, acute toxicities, and preliminary outcomes. International Journal of Radiation Oncology*Biology*Physics*.* 2012;83(5):1419-1424.

[2]. Ares C, Hug EB, Lomax AJ, et al. Effectiveness and safety of spot scanning proton radiation therapy for chordomas and chondrosarcomas of the skull base: First long-term report. International Journal of Radiation Oncology*Biology*Physics*.* 2009;75(4):1111-1118.

[3]. Arimoto T, Kitagawa T, Tsujii H, Ohhara K. High-energy proton beam radiation therapy for gynecologic malignancies. Potential of proton beam as an alternative to brachytherapy. Cancer*.* 1991;68(1):79-83.

[4]. Aziz S, Taylor A, McConnachie A, Kacperek A, Kemp E. Proton beam radiotherapy in the management of uveal melanoma: Clinical experience in scotland. Clinical ophthalmology (Auckland, NZ)*.* 2009;3:49-55.

[5]. Bensoussan E, Thariat J, Maschi C, et al. Outcomes after proton beam therapy for large choroidal melanomas in 492 patients. American Journal Of Ophthalmology*.* 2016;165:78-87.

[6]. Bhattasali OMDMPH, Holliday EMD, Kies MSMD, et al. Definitive proton radiation therapy and concurrent cisplatin for unresectable head and neck adenoid cystic carcinoma: A series of 9 cases and a critical review of the literature. Head & Neck*.* 2016;38 Supplement(1):E1472-E1480.

[7]. Blanchard P, Garden AS, Gunn GB, et al. Intensity-modulated proton beam therapy (impt) versus intensity-modulated photon therapy (imrt) for patients with oropharynx cancer – a case matched analysis. Radiotherapy and Oncology*.* 2016;120(1):48-55.

[8]. Böker A, Pilger D, Cordini D, et al. Neoadjuvant proton beam irradiation vs. Adjuvant ruthenium brachytherapy in transscleral resection of uveal melanoma. Graefe's Archive for Clinical and Experimental Ophthalmology*.* 2018;10.1007/s00417-018-4032-7.

[9]. Bonnet RB, Bush D, Cheek GA, et al. Effects of proton and combined proton/photon beam radiation on pulmonary function in patients with resectable but medically inoperable non-small cell lung cancer. Chest*.* 2001;120(6):1803-1810.

[10]. Boskos C, Feuvret L, Noel G, et al. Combined proton and photon conformal radiotherapy for intracranial atypical and malignant meningioma. International Journal of Radiation Oncology*Biology*Physics*.* 2009;75(2):399-406.

[11]. Brodin NP, Vogelius IR, Maraldo MV, et al. Life years lost--comparing potentially fatal late complications after radiotherapy for pediatric medulloblastoma on a common scale. Cancer*.* 2012;118(21):5432-5440.

[12]. Brown AP, Barney CL, Grosshans DR, et al. Proton beam craniospinal irradiation reduces acute toxicity for adults with medulloblastoma. Int J Radiat Oncol Biol Phys*.* 2013;86(2):277-284.

[13]. Bush DA, Do S, Lum S, et al. Partial breast radiation therapy with proton beam: 5-year results with cosmetic outcomes. International Journal of Radiation Oncology*Biology*Physics*.* 2014;90(3):501-505.

[14]. Bush DA, Kayali Z, Grove R, Slater JD. The safety and efficacy of high-dose proton beam radiotherapy for hepatocellular carcinoma: A phase 2 prospective trial. Cancer*.* 2011;117(13):3053-3059.

[15]. Bush DA, Slater JD, Bonnet R, et al. Proton-beam radiotherapy for early-stage lung cancer. Chest*.* 1999;116(5):1313-1319.

[16]. Bush DA, Slater JD, Garberoglio C, Do S, Lum S, Slater JM. Partial breast irradiation delivered with proton beam: Results of a phase ii trial. Clinical Breast Cancer*.* 2011;11(4):241-245.

[17]. Bush DA, Smith JC, Slater JD, et al. Randomized clinical trial comparing proton beam radiation therapy with transarterial chemoembolization for hepatocellular carcinoma: Results of an interim analysis. Int J Radiat Oncol Biol Phys*.* 2016;95(1):477-482.

[18]. Bush DAMD, Slater JDMD, Shin BBBA, Cheek GMDF, Miller DWP, Slater JMMD. Hypofractionated proton beam radiotherapy for stage i lung cancer*. Chest*.* 2004;126(4):1198-1203.

[19]. Caujolle J-P, Paoli V, Chamorey E, et al. Local recurrence after uveal melanoma proton beam therapy: Recurrence types and prognostic consequences. International Journal Of Radiation Oncology, Biology, Physics*.* 2013;85(5):1218-1224.

[20]. Chang JH, Lee NK, Kim JY, et al. Phase ii trial of proton beam accelerated partial breast irradiation in breast cancer. Radiotherapy and Oncology*.* 2013;108(2):209-214.

[21]. Chang JY, Komaki R, Lu C, et al. Phase 2 study of high-dose proton therapy with concurrent chemotherapy for unresectable stage iii nonsmall cell lung cancer. Cancer*.* 2011;117(20):4707-4713.

[22]. Chang JY, Komaki R, Wen HY, et al. Toxicity and patterns of failure of adaptive/ablative proton therapy for early-stage, medically inoperable non–small cell lung cancer. International Journal of Radiation Oncology*Biology*Physics*.* 2011;80(5):1350-1357.

[23]. Char DH, Kroll S, Phillips TL, Quivey JM. Late radiation failures after iodine 125 brachytherapy for uveal melanoma compared with charged-particle (proton or helium ion) therapy. Ophthalmology*.* 2002;109(10):1850-1854.

[24]. Char DH, Bove R, Phillips TL. Laser and proton radiation to reduce uveal melanoma-associated exudative retinal detachments. Am J Ophthalmol*.* 2003;136(1):180-182.

[25]. Chen Y-LMD, Liebsch NMDP, Kobayashi WBA, et al. Definitive high-dose photon/proton radiotherapy for unresected mobile spine and sacral chordomas. Spine*.* 2013;38(15):E930-E936.

[26]. Chiba T, Tokuuye K, Matsuzaki Y, et al. Proton beam therapy for hepatocellular carcinoma: A retrospective review of 162 patients. Clin Cancer Res*.* 2005;11(10):3799-3805.

[27]. Childs SK, Kozak KR, Friedmann AM, et al. Proton radiotherapy for parameningeal rhabdomyosarcoma: Clinical outcomes and late effects. International Journal of Radiation Oncology*Biology*Physics*.* 2012;82(2):635-642.

[28]. Christopherson K, Werning JW, Malyapa RS, Morris CG, Mendenhall WM. Radiotherapy for sinonasal undifferentiated carcinoma. American Journal of Otolaryngology*.* 2014;35(2):141-146.

[29]. Ciernik IF, Niemierko A, Harmon DC, et al. Proton-based radiotherapy for unresectable or incompletely resected osteosarcoma. Cancer*.* 2011;117(19):4522-4530.

[30]. Coen JJ, Paly JJ, Niemierko A, et al. Long-term quality of life outcome after proton beam monotherapy for localized prostate cancer. International Journal of Radiation Oncology*Biology*Physics*.* 2012;82(2):e201-e209.

[31]. Coen JJ, Zietman AL, Rossi CJ, et al. Comparison of high-dose proton radiotherapy and brachytherapy in localized prostate cancer: A case-matched analysis. Int J Radiat Oncol Biol Phys*.* 2012;82(1):e25-31.

[32]. Coen JJ, Bae K, Zietman AL, et al. Acute and late toxicity after dose escalation to 82 gye using conformal proton radiation for localized prostate cancer: Initial report of american college of radiology phase ii study 03-12. International Journal of Radiation Oncology*Biology*Physics*.* 2011;81(4):1005-1009.

[33]. Colaco RJ, Huh S, Nichols RC, et al. Dosimetric rationale and early experience at ufpti of thoracic proton therapy and chemotherapy in limited-stage small cell lung cancer. Acta Oncologica (Stockholm, Sweden)*.* 2013;52(3):506-513.

[34]. Courdi A, Caujolle J-P, Grange J-D, et al. Results of proton therapy of uveal melanomas treated in nice. International Journal of Radiation Oncology*Biology*Physics*.* 1999;45(1):5-11.

[35]. Cuaron JJ, Chon B, Tsai H, et al. Early toxicity in patients treated with postoperative proton therapy for locally advanced breast cancer. Int J Radiat Oncol Biol Phys*.* 2015;92(2):284-291.

[36]. Damato B, Kacperek A, Chopra M, Campbell IR, Errington RD. Proton beam radiotherapy of choroidal melanoma: The liverpool-clatterbridge experience. International Journal of Radiation Oncology*Biology*Physics*.* 2005;62(5):1405-1411.

[37]. Damato B, Kacperek A, Chopra M, Sheen MA, Campbell IR, Errington RD. Proton beam radiotherapy of iris melanoma. International Journal of Radiation Oncology*Biology*Physics*.* 2005;63(1):109-115.

[38]. DeLaney TFMD, Liebsch NJMDP, Pedlow FXMD, et al. Long-term results of phase ii study of high dose photon/proton radiotherapy in the management of spine chordomas, chondrosarcomas, and other sarcomas. Journal of Surgical Oncology*.* 2014;110(2):115-122.

[39]. Demizu Y, Fujii O, Terashima K, et al. Particle therapy for mucosal melanoma of the head and neck. A single-institution retrospective comparison of proton and carbon ion therapy. Strahlenther Onkol*.* 2014;190(2):186-191.

[40]. Dendale R, Lumbroso-Le Rouic L, Noel G, et al. Proton beam radiotherapy for uveal melanoma: Results of curie institut–orsay proton therapy center (icpo). International Journal of Radiation Oncology*Biology*Physics*.* 2006;65(3):780-787.

[41]. Deraniyagala RL, Yeung D, Mendenhall WM, et al. Proton therapy for skull base chordomas: An outcome study from the university of florida proton therapy institute. Journal of Neurological Surgery Part B, Skull Base*.* 2014;75(1):53-57.

[42]. Desjardins L, Lumbroso-Le Rouic L, Levy-Gabriel C, et al. Treatment of uveal melanoma by accelerated proton beam. Dev Ophthalmol*.* 2012;49:41-57.

[43]. Desjardins L, Lumbroso-Le Rouic L, Levy-Gabriel C, et al. Combined proton beam radiotherapy and transpupillary thermotherapy for large uveal melanomas: A randomized study of 151 patients. Ophthalmic Res*.* 2006;38(5):255-260.

[44]. Duttenhaver JR, Shipley WU, Perrone T, et al. Protons or megavoltage x-rays as boost therapy for patients irradiated for localized prostatic carcinoma. An early phase i/ii comparison. Cancer*.* 1983;51(9):1599-1604.

[45]. Eaton BR, Esiashvili N, Kim S, et al. Clinical outcomes among children with standard-risk medulloblastoma treated with proton and photon radiation therapy: A comparison of disease control and overall survival. Int J Radiat Oncol Biol Phys*.* 2016;94(1):133-138.

[46]. Egger E, Schalenbourg A, Zografos L, et al. Maximizing local tumor control and survival after proton beam radiotherapy of uveal melanoma. International Journal of Radiation Oncology*Biology*Physics*.* 2001;51(1):138-147.

[47]. Egger E, Zografos L, Munkel G, Bohringer T, Bercher L, Chamot L. Results of proton radiotherapy for uveal melanomas. Front Radiat Ther Oncol*.* 1997;30:111-122.

[48]. Fang P, Mick R, Deville C, et al. A case-matched study of toxicity outcomes after proton therapy and intensity-modulated radiation therapy for prostate cancer. Cancer*.* 2015;121(7):1118-1127.

[49]. Feuvret L, Bracci S, Calugaru V, et al. Efficacy and safety of adjuvant proton therapy combined with surgery for chondrosarcoma of the skull base: A retrospective, population-based study. International Journal Of Radiation Oncology, Biology, Physics*.* 2016;95(1):312-321.

[50]. Fitzek MM, Thornton AF, Rabinov JD, et al. Accelerated fractionated proton/photon irradiation to 90 cobalt gray equivalent for glioblastoma multiforme: Results of a phase ii prospective trial. J Neurosurg*.* 1999;91(2):251-260.

[51]. Fitzek MM, Thornton AF, Varvares M, et al. Neuroendocrine tumors of the sinonasal tract. Cancer*.* 2002;94(10):2623-2634.

[52]. Frank SJ, Cox JD, Gillin M, et al. Multifield optimization intensity modulated proton therapy for head and neck tumors: A translation to practice. International Journal of Radiation Oncology*Biology*Physics*.* 2014;89(4):846-853.

[53]. Fuji H, Yoshikawa S, Kasami M, et al. High-dose proton beam therapy for sinonasal mucosal malignant melanoma. Radiation Oncology (London, England)*.* 2014;9:162-162.

[54]. Fujii O, Demizu Y, Hashimoto N, et al. A retrospective comparison of proton therapy and carbon ion therapy for stage i non-small cell lung cancer. Radiother Oncol*.* 2013;109(1):32-37.

[55]. Fujii O, Demizu Y, Hashimoto N, et al. Particle therapy for clinically diagnosed stage i lung cancer: Comparison with pathologically proven non-small cell lung cancer. Acta Oncol*.* 2015;54(3):315-321.

[56]. Fukumitsu N, Okumura T, Mizumoto M, et al. Outcome of t4 (international union against cancer staging system, 7th edition) or recurrent nasal cavity and paranasal sinus carcinoma treated with proton beam. International Journal of Radiation Oncology*Biology*Physics*.* 2012;83(2):704-711.

[57]. Fukumitsu N, Sugahara S, Nakayama H, et al. A prospective study of hypofractionated proton beam therapy for patients with hepatocellular carcinoma. International Journal of Radiation Oncology*Biology*Physics*.* 2009;74(3):831-836.

[58]. Fuss M, Loredo LN, Blacharski PA, Grove RI, Slater JD. Proton radiation therapy for medium and large choroidal melanoma: Preservation of the eye and its functionality. Int J Radiat Oncol Biol Phys*.* 2001;49(4):1053-1059.

[59]. Galland-Girodet S, Pashtan I, MacDonald SM, et al. Long-term cosmetic outcomes and toxicities of proton beam therapy compared with photon-based 3-dimensional conformal accelerated partial-breast irradiation: A phase 1 trial. Int J Radiat Oncol Biol Phys*.* 2014;90(3):493-500.

[60]. Gardner BG, Zietman AL, Shipley WU, Skowronski UE, McManus P. Late normal tissue sequelae in the second decade after high dose radiation therapy with combined photons and conformal protons for locally advanced prostate cancer. J Urol*.* 2002;167(1):123-126.

[61]. Gragoudas EMD, Li WMS, Goitein MP, Lane AMMPH, Munzenrider JEMD, Egan KMD. Evidence-based estimates of outcome in patients irradiated for intraocular melanoma. Archives of Ophthalmology*.* 2002;120(12):1665-1671.

[62]. Gragoudas ES, Lane AM, Regan S, et al. A randomized controlled trial of varying radiation doses in the treatment of choroidal melanoma. Arch Ophthalmol*.* 2000;118(6):773-778.

[63]. Grant SR, Grosshans DR, Bilton SD, et al. Proton versus conventional radiotherapy for pediatric salivary gland tumors: Acute toxicity and dosimetric characteristics. Radiother Oncol*.* 2015;116(2):309-315.

[64]. Gray PJMD, Paly JJBS, Yeap BYS, et al. Patient-reported outcomes after 3-dimensional conformal, intensity-modulated, or proton beam radiotherapy for localized prostate cancer. Cancer*.* 2013;119(9):1729-1735.

[65]. Greenberger BA, Pulsifer MB, Ebb DH, et al. Clinical outcomes and late endocrine, neurocognitive, and visual profiles of proton radiation for pediatric low-grade gliomas. International Journal Of Radiation Oncology, Biology, Physics*.* 2014;89(5):1060-1068.

[66]. Gunn GB, Blanchard P, Garden AS, et al. Clinical outcomes and patterns of disease recurrence after intensity modulated proton therapy for oropharyngeal squamous carcinoma. *International Journal of Radiation Oncology Biology Physics.* 2016;95(1):360-367. <http://onlinelibrary.wiley.com/o/cochrane/clcentral/articles/867/CN-01153867/frame.html>.

[67]. Habl G, Uhl M, Katayama S, et al. Acute toxicity and quality of life in patients with prostate cancer treated with protons or carbon ions in a prospective randomized phase ii study--the ipi trial. Int J Radiat Oncol Biol Phys*.* 2016;95(1):435-443.

[68]. Habrand J-L, Schneider R, Alapetite C, et al. Proton therapy in pediatric skull base and cervical canal low-grade bone malignancies. International Journal of Radiation Oncology*Biology*Physics*.* 2008;71(3):672-675.

[69]. Hashimoto T, Tokuuye K, Fukumitsu N, et al. Repeated proton beam therapy for hepatocellular carcinoma. International Journal of Radiation Oncology*Biology*Physics*.* 2006;65(1):196-202.

[70]. Hata M, Tokuuye K, Kagei K, et al. Hypofractionated high-dose proton beam therapy for stage i non–small-cell lung cancer: Preliminary results of a phase i/ii clinical study. International Journal of Radiation Oncology*Biology*Physics*.* 2007;68(3):786-793.

[71]. Hata M, Tokuuye K, Sugahara S, et al. Proton beam therapy for hepatocellular carcinoma with limited treatment options. Cancer*.* 2006;107(3):591-598.

[72]. Hata M, Tokuuye K, Sugahara S, et al. Proton beam therapy for hepatocellular carcinoma patients with severe cirrhosis. Strahlentherapie und Onkologie*.* 2006;182(12):713.

[73]. Hata M, Tokuuye K, Sugahara S, et al. Proton beam therapy for hepatocellular carcinoma with portal vein tumor thrombus. Cancer*.* 2005;104(4):794-801.

[74]. Hata M, Tokuuye K, Sugahara S, et al. Proton beam therapy for aged patients with hepatocellular carcinoma. International Journal of Radiation Oncology*Biology*Physics*.* 2007;69(3):805-812.

[75]. Hattangadi JA, Rombi B, Yock TI, et al. Proton radiotherapy for high-risk pediatric neuroblastoma: Early outcomes and dose comparison. Int J Radiat Oncol Biol Phys*.* 2012;83(3):1015-1022.

[76]. Hauswald H, Rieken S, Ecker S, et al. First experiences in treatment of low-grade glioma grade i and ii with proton therapy. Radiation Oncology (London, England)*.* 2012;7:189-189.

[77]. Herr MW, Sethi RKV, Meier JC, et al. Esthesioneuroblastoma: An update on the massachusetts eye and ear infirmary and massachusetts general hospital experience with craniofacial resection, proton beam radiation, and chemotherapy. Journal of Neurological Surgery Part B, Skull Base*.* 2014;75(1):58-64.

[78]. Higgins KA, O'Connell K, Liu Y, et al. National cancer database analysis of proton versus photon radiation therapy in non-small cell lung cancer. Int J Radiat Oncol Biol Phys*.* 2017;97(1):128-137.

[79]. Holliday EB, Garden AS, Rosenthal DI, et al. Proton therapy reduces treatment-related toxicities for patients with nasopharyngeal cancer: A case-match control study of intensity-modulated proton therapy and intensity-modulated photon therapy. International Journal of Particle Therapy*.* 2015;2(1):19-28.

[80]. Holliday EB, Mitra HS, Somerson JS, et al. Postoperative proton therapy for chordomas and chondrosarcomas of the spine: Adjuvant versus salvage radiation therapy. Spine (Phila Pa 1976)*.* 2015;40(8):544-549.

[81]. Hoppe BS, Nichols RC, Henderson RH, et al. Erectile function, incontinence, and other quality of life outcomes following proton therapy for prostate cancer in men 60 years old and younger. Cancer*.* 2012;118(18):4619-4626.

[82]. Hoppe BSMDMPH, Michalski JMMD, Mendenhall NPMD, et al. Comparative effectiveness study of patient-reported outcomes after proton therapy or intensity-modulated radiotherapy for prostate cancer. Cancer*.* 2014;120(7):1076-1082.

[83]. Hug EB, Loredo LN, Slater JD, et al. Proton radiation therapy for chordomas and chondrosarcomas of the skull base. J Neurosurg*.* 1999;91(3):432-439.

[84]. Hug EB, Muenter MW, Archambeau JO, et al. Conformal proton radiation therapy for pediatric low-grade astrocytomas. Strahlenther Onkol*.* 2002;178(1):10-17.

[85]. Hug EB, Sweeney RA, Nurre PM, Holloway KC, Slater JD, Munzenrider JE. Proton radiotherapy in management of pediatric base of skull tumors. International Journal of Radiation Oncology*Biology*Physics*.* 2002;52(4):1017-1024.

[86]. Hungerford JL, Foss AJ, Whelahan I, Errington RD, Kacperek A, Kongerud J. Side effects of photon and proton radiotherapy for ocular melanoma. Front Radiat Ther Oncol*.* 1997;30:287-293.

[87]. Igaki H, Tokuuye K, Okumura T, et al. Clinical results of proton beam therapy for skull base chordoma. Int J Radiat Oncol Biol Phys*.* 2004;60(4):1120-1126.

[88]. ISHIKAWA H, HASHIMOTO T, MORIWAKI T, et al. Proton beam therapy combined with concurrent chemotherapy for esophageal cancer. Anticancer Research*.* 2015;35(3):1757-1762.

[89]. Iwata H, Ishikawa H, Takagi M, et al. Long-term outcomes of proton therapy for prostate cancer in japan: A multi-institutional survey of the japanese radiation oncology study group. Cancer medicine*.* 2018;7(3):677-689.

[90]. Iwata H, Murakami M, Demizu Y, et al. High-dose proton therapy and carbon-ion therapy for stage i nonsmall cell lung cancer. Cancer*.* 2010;116(10):2476-2485.

[91]. Iwata HMDP, Demizu YMDP, Fujii OMDP, et al. Long-term outcome of proton therapy and carbon-ion therapy for large (t2a-t2bn0m0) non-small-cell lung cancer. Journal of Thoracic Oncology*.* 2013;8(6):726-735.

[92]. Jimenez RB, Sethi R, Depauw N, et al. Proton radiation therapy for pediatric medulloblastoma and supratentorial primitive neuroectodermal tumors: Outcomes for very young children treated with upfront chemotherapy. International Journal of Radiation Oncology*Biology*Physics*.* 2013;87(1):120-126.

[93]. Kagei K, Tokuuye K, Okumura T, et al. Long-term results of proton beam therapy for carcinoma of the uterine cervix. International Journal of Radiation Oncology*Biology*Physics*.* 2003;55(5):1265-1271.

[94]. Kamran SC, Collier JM, Lane AM, et al. Outcomes of proton therapy for the treatment of uveal metastases. Int J Radiat Oncol Biol Phys*.* 2014;90(5):1044-1050.

[95]. Kanemoto A, Okumura T, Ishikawa H, et al. Outcomes and prognostic factors for recurrence after high-dose proton beam therapy for centrally and peripherally located stage i non--small-cell lung cancer. Clinical Lung Cancer*.* 2014;15(2):e7-e12.

[96]. Kawashima M, Furuse J, Nishio T, et al. Phase ii study of radiotherapy employing proton beam for hepatocellular carcinoma. Journal of Clinical Oncology*.* 2005;23(9):1839-1846.

[97]. Kil WJ, Nichols RC, Jr., Hoppe BS, et al. Hypofractionated passively scattered proton radiotherapy for low- and intermediate-risk prostate cancer is not associated with post-treatment testosterone suppression. Acta Oncol*.* 2013;52(3):492-497.

[98]. Kim IK, Lane AM, Egan KM, Munzenrider J, Gragoudas ES. Natural history of radiation papillopathy after proton beam irradiation of parapapillary melanoma. Ophthalmology*.* 2010;117(8):1617-1622.

[99]. Kim YJ, Cho KH, Pyo HR, et al. A phase ii study of hypofractionated proton therapy for prostate cancer. Acta Oncol*.* 2013;52(3):477-485.

[100]. Komatsu S, Fukumoto T, Demizu Y, et al. Clinical results and risk factors of proton and carbon ion therapy for hepatocellular carcinoma. Cancer*.* 2011;117(21):4890-4904.

[101]. Konstantinidis L, Roberts D, Errington RD, Kacperek A, Heimann H, Damato B. Transpalpebral proton beam radiotherapy of choroidal melanoma. The British Journal Of Ophthalmology*.* 2015;99(2):232-235.

[102]. Koyama S, Tsujii H. Proton beam therapy with high-dose irradiation for superficial and advanced esophageal carcinomas. Clinical Cancer Research*.* 2003;9(10):3571.

[103]. Kozak KR, Smith BL, Adams J, et al. Accelerated partial-breast irradiation using proton beams: Initial clinical experience. International Journal of Radiation Oncology*Biology*Physics*.* 2006;66(3):691-698.

[104]. Kuhlthau KA, Pulsifer MB, Yeap BY, et al. Prospective study of health-related quality of life for children with brain tumors treated with proton radiotherapy. Journal of Clinical Oncology*.* 2012;30(17):2079-2086.

[105]. Ladra MM, Szymonifka JD, Mahajan A, et al. Preliminary results of a phase ii trial of proton radiotherapy for pediatric rhabdomyosarcoma. *Journal of clinical oncology : official journal of the American Society of Clinical Oncology.* 2014;32(33):3762-3770. <http://onlinelibrary.wiley.com/o/cochrane/clcentral/articles/756/CN-01050756/frame.html>.

[106]. Lane AM, Kim IK, Gragoudas ES. Long-term risk of melanoma-related mortality for patients with uveal melanoma treated with proton beam therapy. JAMA Ophthalmology*.* 2015;133(7):792-796.

[107]. Lane AMMPH, Kim IKMD, Gragoudas ESMD. Proton irradiation for peripapillary and parapapillary melanomas. Archives of Ophthalmology*.* 2011;129(9):1127-1130.

[108]. Leiser D, Calaminus G, Malyapa R, et al. Tumour control and quality of life in children with rhabdomyosarcoma treated with pencil beam scanning proton therapy. Radiotherapy and Oncology*.* 2016;120(1):163-168.

[109]. Lewis GDMD, Holliday EBMD, Kocak-Uzel EMD, et al. Intensity-modulated proton therapy for nasopharyngeal carcinoma: Decreased radiation dose to normal structures and encouraging clinical outcomes. Head & Neck*.* 2016;38 Supplement(1):E1886-E1895.

[110]. Liao Z, Lee JJ, Komaki R, et al. Bayesian adaptive randomization trial of passive scattering proton therapy and intensity-modulated photon radiotherapy for locally advanced non–small-cell lung cancer. Journal of Clinical Oncology*.* 2018;36(18):1813-1822.

[111]. Lin LL, Kirk M, Scholey J, et al. Initial report of pencil beam scanning proton therapy for posthysterectomy patients with gynecologic cancer. Int J Radiat Oncol Biol Phys*.* 2016;95(1):181-189.

[112]. Lin SH, Komaki R, Liao Z, et al. Proton beam therapy and concurrent chemotherapy for esophageal cancer. International Journal Of Radiation Oncology, Biology, Physics*.* 2012;83(3):e345-e351.

[113]. Linton OR, Moore MG, Brigance JS, Summerlin D-J, McDonald MW. Proton therapy for head and neck adenoid cystic carcinoma: Initial clinical outcomes. Head & Neck*.* 2015;37(1):117-124.

[114]. Lucas JT, Jr., Ladra MM, MacDonald SM, et al. Proton therapy for pediatric and adolescent esthesioneuroblastoma. Pediatric Blood & Cancer*.* 2015;62(9):1523-1528.

[115]. Lumbroso-Le Rouic L, Delacroix S, Dendale R, et al. Proton beam therapy for iris melanomas. Eye (Lond)*.* 2006;20(11):1300-1305.

[116]. Macdonald EC, Cauchi P, Kemp EG. Proton beam therapy for the treatment of uveal melanoma in scotland. Br J Ophthalmol*.* 2011;95(12):1691-1695.

[117]. MacDonald SM, Patel SA, Hickey S, et al. Proton therapy for breast cancer after mastectomy: Early outcomes of a prospective clinical trial. Int J Radiat Oncol Biol Phys*.* 2013;86(3):484-490.

[118]. MacDonald SM, Sethi R, Lavally B, et al. Proton radiotherapy for pediatric central nervous system ependymoma: Clinical outcomes for 70 patients. Neuro-Oncology*.* 2013;15(11):1552-1559.

[119]. Makita C, Nakamura T, Takada A, et al. Clinical outcomes and toxicity of proton beam therapy for advanced cholangiocarcinoma. Radiation Oncology (London, England)*.* 2014;9:26-26.

[120]. Maquilan G, Grover S, Alonso-Basanta M, Lustig RA. Acute toxicity profile of patients with low-grade gliomas and meningiomas receiving proton therapy. Am J Clin Oncol*.* 2014;37(5):438-443.

[121]. Marucci L, Ancukiewicz M, Lane AM, Collier JM, Gragoudas ES, Munzenrider JE. Uveal melanoma recurrence after fractionated proton beam therapy: Comparison of survival in patients treated with reirradiation or with enucleation. Int J Radiat Oncol Biol Phys*.* 2011;79(3):842-846.

[122]. Matsuzaki Y, Osuga T, Saito Y, et al. A new, effective, and safe therapeutic option using proton irradiation for hepatocellular carcinoma. Gastroenterology*.* 1994;106(4):1032-1041.

[123]. McAvoy SA, Ciura KT, Rineer JM, et al. Feasibility of proton beam therapy for reirradiation of locoregionally recurrent non-small cell lung cancer. Radiotherapy And Oncology: Journal Of The European Society For Therapeutic Radiology And Oncology*.* 2013;109(1):38-44.

[124]. McDonald MW, Liu Y, Moore MG, Johnstone PAS. Acute toxicity in comprehensive head and neck radiation for nasopharynx and paranasal sinus cancers: Cohort comparison of 3d conformal proton therapy and intensity modulated radiation therapy. Radiation Oncology (London, England)*.* 2016;11:32-32.

[125]. McDonald MW, Linton OR, Shah MV. Proton therapy for reirradiation of progressive or recurrent chordoma. Int J Radiat Oncol Biol Phys*.* 2013;87(5):1107-1114.

[126]. McGee L, Mendenhall NP, Henderson RH, et al. Outcomes in men with large prostates (≥ 60 cm(3)) treated with definitive proton therapy for prostate cancer. Acta Oncologica (Stockholm, Sweden)*.* 2013;52(3):470-476.

[127]. McGovern SL, Okcu MF, Munsell MF, et al. Outcomes and acute toxicities of proton therapy for pediatric atypical teratoid/rhabdoid tumor of the central nervous system. International Journal Of Radiation Oncology, Biology, Physics*.* 2014;90(5):1143-1152.

[128]. Mendenhall NP, Li Z, Hoppe BS, et al. Early outcomes from three prospective trials of image-guided proton therapy for prostate cancer. Int J Radiat Oncol Biol Phys*.* 2012;82(1):213-221.

[129]. Mendenhall NP, Hoppe BS, Nichols RC, et al. Five-year outcomes from 3 prospective trials of image-guided proton therapy for prostate cancer. Int J Radiat Oncol Biol Phys*.* 2014;88(3):596-602.

[130]. Mizumoto M, Sugahara S, Nakayama H, et al. Clinical results of proton-beam therapy for locoregionally advanced esophageal cancer. Strahlentherapie Und Onkologie: Organ Der Deutschen Rontgengesellschaft [Et Al]*.* 2010;186(9):482-488.

[131]. Mizumoto M, Tsuboi K, Igaki H, et al. Phase i/ii trial of hyperfractionated concomitant boost proton radiotherapy for supratentorial glioblastoma multiforme. International Journal of Radiation Oncology*Biology*Physics*.* 2010;77(1):98-105.

[132]. Moeller BJ, Chintagumpala M, Philip JJ, et al. Low early ototoxicity rates for pediatric medulloblastoma patients treated with proton radiotherapy. Radiation Oncology*.* 2011;6(1):58.

[133]. Morimoto K, Demizu Y, Hashimoto N, et al. Particle radiotherapy using protons or carbon ions for unresectable locally advanced head and neck cancers with skull base invasion. Jpn J Clin Oncol*.* 2014;44(5):428-434.

[134]. Mosci C, Mosci S, Barla A, Squarcia S, Chauvel P, Iborra N. Proton beam radiotherapy of uveal melanoma: Italian patients treated in nice, france. Eur J Ophthalmol*.* 2009;19(4):654-660.

[135]. Mouw KW, Sethi RV, Yeap BY, et al. Proton radiation therapy for the treatment of retinoblastoma. International Journal Of Radiation Oncology, Biology, Physics*.* 2014;90(4):863-869.

[136]. Naeser P, Blomquist E, Montelius A, Thoumas KA. Proton irradiation of malignant uveal melanoma. A five year follow-up of patients treated in uppsala, sweden. Ups J Med Sci*.* 1998;103(3):203-211.

[137]. Nakamura N, Zenda S, Tahara M, et al. Proton beam therapy for olfactory neuroblastoma. Radiotherapy and Oncology*.* 2017;122(3):368-372.

[138]. Nakayama H, Satoh H, Sugahara S, et al. Proton beam therapy of stage ii and iii non–small-cell lung cancer. International Journal of Radiation Oncology*Biology*Physics*.* 2011;81(4):979-984.

[139]. Nakayama H, Sugahara S, Tokita M, et al. Proton beam therapy for patients with medically inoperable stage i non-small-cell lung cancer at the university of tsukuba. Int J Radiat Oncol Biol Phys*.* 2010;78(2):467-471.

[140]. Nakayama HMDP, Sugahara SMDP, Tokita MBA, et al. Proton beam therapy for hepatocellular carcinoma: The university of tsukuba experience. Cancer*.* 2009;115(23):5499-5506.

[141]. Nguyen Q-N, Ly NB, Komaki R, et al. Long-term outcomes after proton therapy, with concurrent chemotherapy, for stage ii–iii inoperable non-small cell lung cancer. Radiotherapy and Oncology*.* 2015;115(3):367-372.

[142]. Nichols RC, Jr., George TJ, Zaiden RA, Jr., et al. Proton therapy with concomitant capecitabine for pancreatic and ampullary cancers is associated with a low incidence of gastrointestinal toxicity. Acta Oncol*.* 2013;52(3):498-505.

[143]. Nichols RC, Jr., Morris CG, Hoppe BS, et al. Proton radiotherapy for prostate cancer is not associated with post-treatment testosterone suppression. Int J Radiat Oncol Biol Phys*.* 2012;82(3):1222-1226.

[144]. Nihei K, Ogino T, Ishikura S, et al. Phase ii feasibility study of high-dose radiotherapy for prostate cancer using proton boost therapy: First clinical trial of proton beam therapy for prostate cancer in japan. Japanese Journal Of Clinical Oncology*.* 2005;35(12):745-752.

[145]. Nihei K, Ogino T, Ishikura S, Nishimura H. High-dose proton beam therapy for stage i non–small-cell lung cancer. International Journal of Radiation Oncology*Biology*Physics*.* 2006;65(1):107-111.

[146]. Nihei K, Ogino T, Onozawa M, et al. Multi-institutional phase ii study of proton beam therapy for organ-confined prostate cancer focusing on the incidence of late rectal toxicities. Int J Radiat Oncol Biol Phys*.* 2011;81(2):390-396.

[147]. Nishimura H, Ogino T, Kawashima M, et al. Proton-beam therapy for olfactory neuroblastoma. Int J Radiat Oncol Biol Phys*.* 2007;68(3):758-762.

[148]. Noel G, Habrand JL, Jauffret E, et al. Radiation therapy for chordoma and chondrosarcoma of the skull base and the cervical spine. Prognostic factors and patterns of failure. Strahlenther Onkol*.* 2003;179(4):241-248.

[149]. Noël G, Feuvret L, Calugaru V, et al. Chordomas of the base of the skull and upper cervical spine. One hundred patients irradiated by a 3d conformal technique combining photon and proton beams. Acta Oncologica*.* 2005;44(7):700-708.

[150]. Ohkawa A, Mizumoto M, Ishikawa H, et al. Proton beam therapy for unresectable intrahepatic cholangiocarcinoma. Journal of Gastroenterology and Hepatology*.* 2015;30(5):957-963.

[151]. Okano S, Tahara M, Zenda S, et al. Induction chemotherapy with docetaxel, cisplatin and s-1 followed by proton beam therapy concurrent with cisplatin in patients with t4b nasal and sinonasal malignancies. Japanese Journal Of Clinical Oncology*.* 2012;42(8):691-696.

[152]. Patel AV, Lane AM, Morrison MA, et al. Visual outcomes after proton beam irradiation for choroidal melanomas involving the fovea. Ophthalmology*.* 2016;123(2):369-377.

[153]. Phan J, Sio TT, Nguyen TP, et al. Reirradiation of head and neck cancers with proton therapy: Outcomes and analyses. International Journal of Radiation Oncology*Biology*Physics*.* 2016;96(1):30-41.

[154]. Pommier P, Liebsch NJ, Deschler DG, et al. Proton beam radiation therapy for skull base adenoid cystic carcinoma. Arch Otolaryngol Head Neck Surg*.* 2006;132(11):1242-1249.

[155]. Pugh TJ, Choi S, Nogueras-Gonzalaez GM, et al. Proton beam therapy for localized prostate cancer: Results from a prospective quality-of-life trial. International Journal of Particle Therapy*.* 2016;3(1):27-36.

[156]. Rahmi A, Mammar H, Thariat J, et al. Proton beam therapy for presumed and confirmed iris melanomas: A review of 36 cases. Graefes Arch Clin Exp Ophthalmol*.* 2014;252(9):1515-1521.

[157]. Remick JS, Schonewolf C, Gabriel P, et al. First clinical report of proton beam therapy for postoperative radiotherapy for non–small-cell lung cancer. Clinical Lung Cancer*.* 2017;18(4):364-371.

[158]. Rich TA, Schiller A, Suit HD, Mankin HJ. Clinical and pathologic review of 48 cases of chordoma. Cancer*.* 1985;56(1):182-187.

[159]. Riechardt AI, Cordini D, Dobner B, et al. Salvage proton beam therapy in local recurrent uveal melanoma. American Journal Of Ophthalmology*.* 2014;158(5):948-956.

[160]. Rombi B, DeLaney TF, MacDonald SM, et al. Proton radiotherapy for pediatric ewing's sarcoma: Initial clinical outcomes. Int J Radiat Oncol Biol Phys*.* 2012;82(3):1142-1148.

[161]. Rombi B, Ares C, Hug EB, et al. Spot-scanning proton radiation therapy for pediatric chordoma and chondrosarcoma: Clinical outcome of 26 patients treated at paul scherrer institute. International Journal of Radiation Oncology*Biology*Physics*.* 2013;86(3):578-584.

[162]. Romesser PB, Cahlon O, Scher ED, et al. Proton beam reirradiation for recurrent head and neck cancer: Multi-institutional report on feasibility and early outcomes. International Journal Of Radiation Oncology, Biology, Physics*.* 2016;95(1):386-395.

[163]. Rossi CJ. Conformal proton beam therapy of prostate cancer--update on the loma linda university medical center experience. Strahlenther Onkol*.* 1999;175 Suppl 2:82-84.

[164]. Rotondo RL, Folkert W, Liebsch NJ, et al. High-dose proton-based radiation therapy in the management of spine chordomas: Outcomes and clinicopathological prognostic factors. J Neurosurg Spine*.* 2015;23(6):788-797.

[165]. Rundle P, Singh AD, Rennie I. Proton beam therapy for iris melanoma: A review of 15 cases. Eye (Lond)*.* 2007;21(1):79-82.

[166]. Rwigema J-CM, Verma V, Lin L, et al. Prospective study of proton-beam radiation therapy for limited-stage small cell lung cancer. Cancer*.* 2017;123(21):4244-4251.

[167]. Sachsman S, Flampouri S, Li Z, Lynch J, Mendenhall NP, Hoppe BS. Proton therapy in the management of non-hodgkin lymphoma. Leuk Lymphoma*.* 2015;56(9):2608-2612.

[168]. Sachsman S, R. Charles Nichols J, Morris CG, et al. Proton therapy and concomitant capecitabine for non-metastatic unresectable pancreatic adenocarcinoma. International Journal of Particle Therapy*.* 2014;1(3):692-701.

[169]. Sandinha MT, Kacperek A, Errington RD, Coupland SE, Damato B. Recurrence of iris melanoma after proton beam therapy. The British Journal Of Ophthalmology*.* 2014;98(4):484-487.

[170]. Schlienger P, Habrand JL, Schwartz L, et al. Initial results with one-year minimum follow-up of the first 146 patients with a uveal melanoma treated with protons at cpo (orsay). Bull Cancer Radiother*.* 1996;83 Suppl:212s-214s.

[171]. Schönfeld S, Cordini D, Riechardt AI, et al. Proton beam therapy leads to excellent local control rates in choroidal melanoma in the intermediate fundus zone. American Journal Of Ophthalmology*.* 2014;158(6):1184-1191.

[172]. Seddon JM, Gragoudas ES, Albert DM, Hsieh CC, Polivogianis L, Friedenberg GR. Comparison of survival rates for patients with uveal melanoma after treatment with proton beam irradiation or enucleation. Am J Ophthalmol*.* 1985;99(3):282-290.

[173]. Seddon JM, Gragoudas ES, Egan KM, et al. Relative survival rates after alternative therapies for uveal melanoma. Ophthalmology*.* 1990;97(6):769-777.

[174]. Seibel I, Cordini D, Hager A, et al. Cataract development in patients treated with proton beam therapy for uveal melanoma. Graefe's Archive For Clinical And Experimental Ophthalmology = Albrecht Von Graefes Archiv Fur Klinische Und Experimentelle Ophthalmologie*.* 2016;254(8):1625-1630.

[175]. Seibel I, Cordini D, Rehak M, et al. Local recurrence after primary proton beam therapy in uveal melanoma: Risk factors, retreatment approaches, and outcome. American Journal Of Ophthalmology*.* 2015;160(4):628-636.

[176]. Sejpal S, Komaki R, Tsao A, et al. Early findings on toxicity of proton beam therapy with concurrent chemotherapy for nonsmall cell lung cancer. *Cancer.* 2011;117(13):3004-3013.

[177]. Sethi RV, Shih HA, Yeap BY, et al. Second nonocular tumors among survivors of retinoblastoma treated with contemporary photon and proton radiotherapy. Cancer*.* 2014;120(1):126-133.

[178]. Sherman JC, Colvin MK, Mancuso SM, et al. Neurocognitive effects of proton radiation therapy in adults with low-grade glioma. Journal of Neuro-Oncology*.* 2016;126(1):157-164.

[179]. Shioyama Y, Tokuuye K, Okumura T, et al. Clinical evaluation of proton radiotherapy for non–small-cell lung cancer. International Journal of Radiation Oncology*Biology*Physics*.* 2003;56(1):7-13.

[180]. Shipley WU, Tepper JE, Prout GR, Jr, et al. Proton radiation as boost therapy for localized prostatic carcinoma. JAMA*.* 1979;241(18):1912-1915.

[181]. Shipley WU, Verhey LJ, Munzenrider JE, et al. Advanced prostate cancer: The results of a randomized comparative trial of high dose irradiation boosting with conformal protons compared with conventional dose irradiation using photons alone. *International Journal Of Radiation Oncology, Biology, Physics.* 1995;32(1):3-12.

[182]. Sikuade MJ, Salvi S, Rundle PA, Errington DG, Kacperek A, Rennie IG. Outcomes of treatment with stereotactic radiosurgery or proton beam therapy for choroidal melanoma. Eye (Lond)*.* 2015;29(9):1194-1198.

[183]. Sio TT, Lin HK, Shi Q, et al. Intensity modulated proton therapy versus intensity modulated photon radiation therapy for oropharyngeal cancer: First comparative results of patient-reported outcomes. Int J Radiat Oncol Biol Phys*.* 2016;95(4):1107-1114.

[184]. Slater JD, Austin-Seymour M, Munzenrider J, et al. Endocrine function following high dose proton therapy for tumors of the upper clivus. Int J Radiat Oncol Biol Phys*.* 1988;15(3):607-611.

[185]. Slater JD, Rossi CJ, Yonemoto LT, et al. Proton therapy for prostate cancer: The initial loma linda university experience. International Journal of Radiation Oncology Biology Physics*.* 2004;59(2):348-352.

[186]. Slater JD, Yonemoto LT, Mantik DW, et al. Proton radiation for treatment of cancer of the oropharynx: Early experience at loma linda university medical center using a concomitant boost technique. International Journal of Radiation Oncology Biology Physics*.* 2005;62(2):494-500.

[187]. Song S, Park HJ, Yoon JH, et al. Proton beam therapy reduces the incidence of acute haematological and gastrointestinal toxicities associated with craniospinal irradiation in pediatric brain tumors. Acta Oncol*.* 2014;53(9):1158-1164.

[188]. Sugahara S, Nakayama H, Fukuda K, et al. Proton-beam therapy for hepatocellular carcinoma associated with portal vein tumor thrombosis. Strahlenther Onkol*.* 2009;185(12):782-788.

[189]. Sugahara S, Oshiro Y, Nakayama H, et al. Proton beam therapy for large hepatocellular carcinoma. International Journal of Radiation Oncology Biology Physics*.* 2010;76(2):460-466.

[190]. Sugahara S, Tokuuye K, Okumura T, et al. Clinical results of proton beam therapy for cancer of the esophagus. International Journal of Radiation Oncology*Biology*Physics*.* 2005;61(1):76-84.

[191]. Suneja G, Poorvu PD, Hill-Kayser C, Lustig RA. Acute toxicity of proton beam radiation for pediatric central nervous system malignancies. Pediatr Blood Cancer*.* 2013;60(9):1431-1436.

[192]. Takaoka E-I, Miyazaki J, Ishikawa H, et al. Long-term single-institute experience with trimodal bladder-preserving therapy with proton beam therapy for muscle-invasive bladder cancer. Japanese Journal Of Clinical Oncology*.* 2017;47(1):67-73.

[193]. Talcott JA, Rossi C, Shipley WU, et al. Patient-reported long-term outcomes after conventional and high-dose combined proton and photon radiation for early prostate cancer. JAMA*.* 2010;303(11):1046-1053.

[194]. Terashima K, Demizu Y, Hashimoto N, et al. A phase i/ii study of gemcitabine-concurrent proton radiotherapy for locally advanced pancreatic cancer without distant metastasis. Radiotherapy and Oncology*.* 2012;103(1):25-31.

[195]. Timmermann B, Schuck A, Niggli F, et al. Spot-scanning proton therapy for malignant soft tissue tumors in childhood: First experiences at the paul scherrer institute. International Journal of Radiation Oncology*Biology*Physics*.* 2007;67(2):497-504.

[196]. Tokuuye K, Akine Y, Kagei K, et al. Proton therapy for head and neckmalignancies at tsukuba. Strahlentherapie und Onkologie*.* 2004;180(2):96-101.

[197]. Tran E, Ma R, Paton K, Blackmore E, Pickles T. Outcomes of proton radiation therapy for peripapillary choroidal melanoma at the bc cancer agency. Int J Radiat Oncol Biol Phys*.* 2012;83(5):1425-1431.

[198]. Truong MT, Kamat UR, Liebsch NJ, et al. Proton radiation therapy for primary sphenoid sinus malignancies: Treatment outcome and prognostic factors. Head & Neck*.* 2009;31(10):1297-1308.

[199]. Tsina EK, Lane AM, Zacks DN, Munzenrider JE, Collier JM, Gragoudas ES. Treatment of metastatic tumors of the choroid with proton beam irradiation. Ophthalmology*.* 2005;112(2):337-343.

[200]. Vargas CE, Hartsell WF, Dunn M, et al. Image-guided hypofractionated proton beam therapy for low-risk prostate cancer: Analysis of quality of life and toxicity, pcg gu 002. Reports of Practical Oncology & Radiotherapy*.* 2016;21(3):207-212.

[201]. Vavvas D, Kim I, Lane AM, Chaglassian A, Mukai S, Gragoudas E. Posterior uveal melanoma in young patients treated with proton beam therapy. Retina*.* 2010;30(8):1267-1271.

[202]. Viswanathan V, Pradhan KR, Eugster EA. Pituitary hormone dysfunction after proton beam radiation therapy in children with brain tumors. Endocr Pract*.* 2011;17(6):891-896.

[203]. Weber DC, Badiyan S, Malyapa R, et al. Long-term outcomes and prognostic factors of skull-base chondrosarcoma patients treated with pencil-beam scanning proton therapy at the paul scherrer institute. Neuro-Oncology*.* 2016;18(2):236-243.

[204]. Weber DC, Chan AW, Lessell S, et al. Visual outcome of accelerated fractionated radiation for advanced sinonasal malignancies employing photons/protons. Radiotherapy and Oncology*.* 2006;81(3):243-249.

[205]. Weber DC, Rutz HP, Pedroni ES, et al. Results of spot-scanning proton radiation therapy for chordoma and chondrosarcoma of the skull base: The paul scherrer institut experience. International Journal of Radiation Oncology*Biology*Physics*.* 2005;63(2):401-409.

[206]. Weber DC, Ares C, Malyapa R, et al. Tumor control and qol outcomes of very young children with atypical teratoid/rhabdoid tumor treated with focal only chemo-radiation therapy using pencil beam scanning proton therapy. Journal of Neuro-Oncology*.* 2015;121(2):389-397.

[207]. Westover KD, Seco J, Adams JA, et al. Proton sbrt for medically inoperable stage i nsclc. J Thorac Oncol*.* 2012;7(6):1021-1025.

[208]. Willerding GD, Cordini D, Moser L, Krause L, Foerster MH, Bechrakis NE. Neoadjuvant proton beam irradiation followed by transscleral resection of uveal melanoma in 106 cases. The British Journal Of Ophthalmology*.* 2016;100(4):463-467.

[209]. Wilson MW, Hungerford JL. Comparison of episcleral plaque and proton beam radiation therapy for the treatment of choroidal melanoma. Ophthalmology*.* 1999;106(8):1579-1587.

[210]. Yasuda M, Bresson D, Chibbaro S, et al. Chordomas of the skull base and cervical spine: Clinical outcomes associated with a multimodal surgical resection combined with proton-beam radiation in 40 patients. Neurosurg Rev*.* 2012;35(2):171-182; discussion 182-173.

[211]. Yock T, Yeap B, Ebb D, et al. A phase ii trial of proton radiotherapy for medulloblastoma: Preliminary results. J Clin Oncol*.* 2010;28.

[212]. Yock TI, Yeap BY, Ebb DH, et al. Long-term toxic effects of proton radiotherapy for paediatric medulloblastoma: A phase 2 single-arm study. Lancet Oncol*.* 2016;17(3):287-298.

[213]. Yonemoto LT, Slater JD, Rossi CJ, et al. Combined proton and photon conformal radiation therapy for locally advanced carcinoma of the prostate: Preliminary results of a phase iii study. International Journal of Radiation Oncology*Biology*Physics*.* 1997;37(1):21-29.

[214]. Yu JB, Soulos PR, Herrin J, et al. Proton versus intensity-modulated radiotherapy for prostate cancer: Patterns of care and early toxicity. J Natl Cancer Inst*.* 2013;105(1):25-32.

[215]. Yuh GE, Loredo LN, Yonemoto LT, et al. Reducing toxicity from craniospinal irradiation: Using proton beams to treat medulloblastoma in young children. The Cancer Journal*.* 2004;10(6):386-390.

[216]. Zenda S, Kawashima M, Arahira S, et al. Late toxicity of proton beam therapy for patients with the nasal cavity, para-nasal sinuses, or involving the skull base malignancy: Importance of long-term follow-up. International Journal Of Clinical Oncology*.* 2015;20(3):447-454.

[217]. Zenda S, Kawashima M, Nishio T, et al. Proton beam therapy as a nonsurgical approach to mucosal melanoma of the head and neck: A pilot study. International Journal of Radiation Oncology*Biology*Physics*.* 2011;81(1):135-139.

[218]. Zenda S, Kohno R, Kawashima M, et al. Proton beam therapy for unresectable malignancies of the nasal cavity and paranasal sinuses. International Journal of Radiation Oncology*Biology*Physics*.* 2011;81(5):1473-1478.

[219]. Zietman AL, Bae K, Slater JD, et al. Randomized trial comparing conventional-dose with high-dose conformal radiation therapy in early-stage adenocarcinoma of the prostate: Long-term results from proton radiation oncology group/american college of radiology 95-09. J Clin Oncol*.* 2010;28(7):1106-1111.
